# Cyclin G1 induces maladaptive proximal tubule cell dedifferentiation and renal fibrosis through CDK5 activation

**DOI:** 10.1172/JCI158096

**Published:** 2022-12-01

**Authors:** Kensei Taguchi, Bertha C. Elias, Sho Sugahara, Snehal Sant, Benjamin S. Freedman, Sushrut S. Waikar, Ambra Pozzi, Roy Zent, Raymond C. Harris, Samir M. Parikh, Craig R. Brooks

**Affiliations:** 1 Division of Nephrology and Hypertension, Vanderbilt University Medical Center, Nashville, Tennessee, USA.; 2 Kidney Research Institute, Institute for Stem Cell and Regenerative Medicine, and Department of Medicine, Division of Nephrology, University of Washington, Seattle, Washington, USA.; 3 Section of Nephrology, Boston University School of Medicine and Boston Medical Center, Boston, Massachusetts, USA.; 4 Veterans Affairs Hospital, Nashville, Tennessee, USA.; 5 Division of Nephrology, Department of Internal Medicine, Department of Pharmacology, University of Texas Southwestern Medical Center, Dallas, Texas, USA.; 6 Department of Cell and Developmental Biology, Vanderbilt University, Nashville, Tennessee, USA.

**Keywords:** Nephrology, Cell cycle, Chronic kidney disease, Fibrosis

## Abstract

Acute kidney injury (AKI) occurs in approximately 13% of hospitalized patients and predisposes patients to chronic kidney disease (CKD) through the AKI-to-CKD transition. Studies from our laboratory and others have demonstrated that maladaptive repair of proximal tubule cells (PTCs), including induction of dedifferentiation, G_2_/M cell cycle arrest, senescence, and profibrotic cytokine secretion, is a key process promoting AKI-to-CKD transition, kidney fibrosis, and CKD progression. The molecular mechanisms governing maladaptive repair and the relative contribution of dedifferentiation, G_2_/M arrest, and senescence to CKD remain to be resolved. We identified cyclin G1 (CG1) as a factor upregulated in chronically injured and maladaptively repaired PTCs. We demonstrated that global deletion of CG1 inhibits G_2_/M arrest and fibrosis. Pharmacological induction of G_2_/M arrest in CG1-knockout mice, however, did not fully reverse the antifibrotic phenotype. Knockout of CG1 did not alter dedifferentiation and proliferation in the adaptive repair response following AKI. Instead, CG1 specifically promoted the prolonged dedifferentiation of kidney tubule epithelial cells observed in CKD. Mechanistically, CG1 promotes dedifferentiation through activation of cyclin-dependent kinase 5 (CDK5). Deletion of CDK5 in kidney tubule cells did not prevent G_2_/M arrest but did inhibit dedifferentiation and fibrosis. Thus, CG1 and CDK5 represent a unique pathway that regulates maladaptive, but not adaptive, dedifferentiation, suggesting they could be therapeutic targets for CKD.

## Graphical abstract

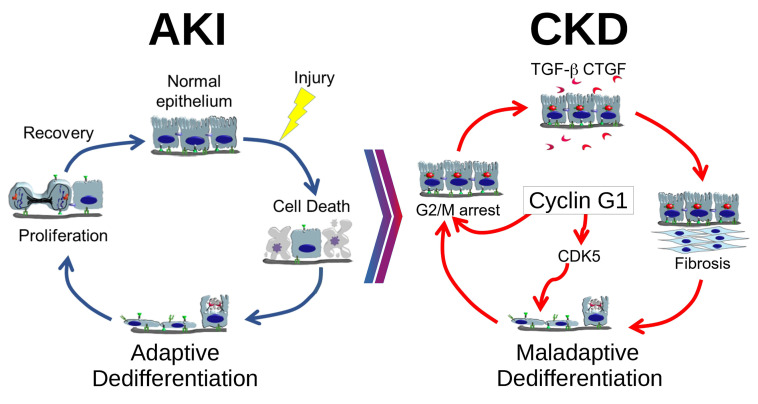

## Introduction

Acute kidney injury (AKI) occurs in approximately 13% of hospitalized patients and is associated with a 4-fold increase in mortality (1). Proximal tubule cells (PTCs) are particularly sensitive to acute injury, accounting for most of the cell death and injury following AKI (2). Following injury, PTCs have the unique ability to divide and replace the damaged epithelium, resulting in a complete recovery of renal function in most cases (2). The kidney has a large renal functional reserve, however, often masking underlying persistent injury following AKI, which can develop into kidney fibrosis and functional decline (3, 4). Thus, an episode of AKI can predispose patients to develop chronic kidney disease (CKD), a progressive disease that affects approximately 10% of the global population and often develops into end-stage renal disease, requiring renal replacement therapy.

Recovery from AKI largely involves repopulation of the lost tubule cells by existing tubule epithelial cells (5–7). In order to achieve this recovery, the remaining tubule cells undergo dedifferentiation, a process whereby surviving tubule cells reversibly revert to an earlier developmental state. This allows the cells to change morphology, enter the cell cycle, and divide to cover areas of the basement membrane exposed by cell loss. Once the tubule is repopulated with cells, the PTCs redifferentiate to their original state (3, 7). Dedifferentiation and expression of cell cycle markers is also a prominent feature of AKI-to-CKD transition and fibrosis progression. Though the kidney tubules appear largely recovered histologically, PTCs continue to attempt cell division but are often arrested at the G_2_/M transition marked by the phosphorylation of histone H3 (p-H3) (3, 8–10). This phenomenon has been referred to as “maladaptive repair” or “failed repair” (9, 11–13). Maladaptively repaired cells actively promote fibrosis progression through the secretion of profibrotic factors (connective tissue growth factor [CTGF], TGF-β, etc.) and increased production of extracellular matrix proteins (9).

Recent studies using single-nucleus RNA sequencing (snRNAseq) have confirmed that a dedifferentiated population of PTCs contributes to CKD but failed to demonstrate an association with G_2_/M arrest (12–14). While this could be a limitation of the snRNAseq approach, as the nuclear membrane breaks down in late G_2_ into M phase, releasing RNA, it raises questions about the relative contribution of dedifferentiation versus G_2_/M arrest in the progression of CKD and whether dedifferentiation following AKI is the same as observed in maladaptively repaired PTCs. Since the key factors regulating PTC G_2_/M arrest and dedifferentiation are largely unknown, these questions have been difficult to address experimentally.

Recently, we identified an atypical cyclin, cyclin G1 (CG1), that is expressed in chronically injured PTCs (15). The mechanism by which CG1 promotes CKD is yet to be defined. CG1 is not necessary for cell cycle progression and CG1-knockout (CG1-KO) mice develop normally but have increased sensitivity to γ radiation (16). In the kidney, CG1 is minimally expressed under basal conditions but is dramatically upregulated in models of CKD (15). In cancer studies, CG1 has been shown to act through activation of its cyclin-dependent kinase 5 (CDK5) (17–19) or through direct interaction with cell cycle regulators, such as p53 (17, 20). CDK5 is known to regulate cell cycle exit in terminally differentiated cells, such as neurons and podocytes, and its activity is normally modulated by p35 (21–23). While not normally expressed in kidney tubular cells, both CDK5 and p35 are upregulated in kidney tubule epithelial cells following injury (24). In the case of cellular stress, cleavage of p35 to p25 induces a hyperactivation of CDK5 that can trigger cell cycle reentry in neurons, followed by G_2_/M arrest and neurodegeneration (22). The roles of CG1 and its downstream factors CDK5 and p35 in CKD have yet to be established.

In this study, we sought to determine the relationship between dedifferentiation and G_2_/M arrest as well as the contribution of each to kidney fibrosis in CKD by targeting CG1. We examined the role of CG1 in fibrosis progression in several models of CKD. We tested whether deletion or inhibition of CG1 or its downstream factors can elucidate the interplay of G_2_/M arrest, dedifferentiation, and maladaptive repair as well as their contributions to kidney fibrosis. We also examined the effects of pharmacological G_2_/M induction on fibrosis downstream of CG1. The therapeutic potential of targeting the CG1 pathway was examined through the pharmacological inhibition or conditional deletion of CDK5.

## Results

### CG1 promotes renal fibrosis in models of CKD.

**Figure 1:**
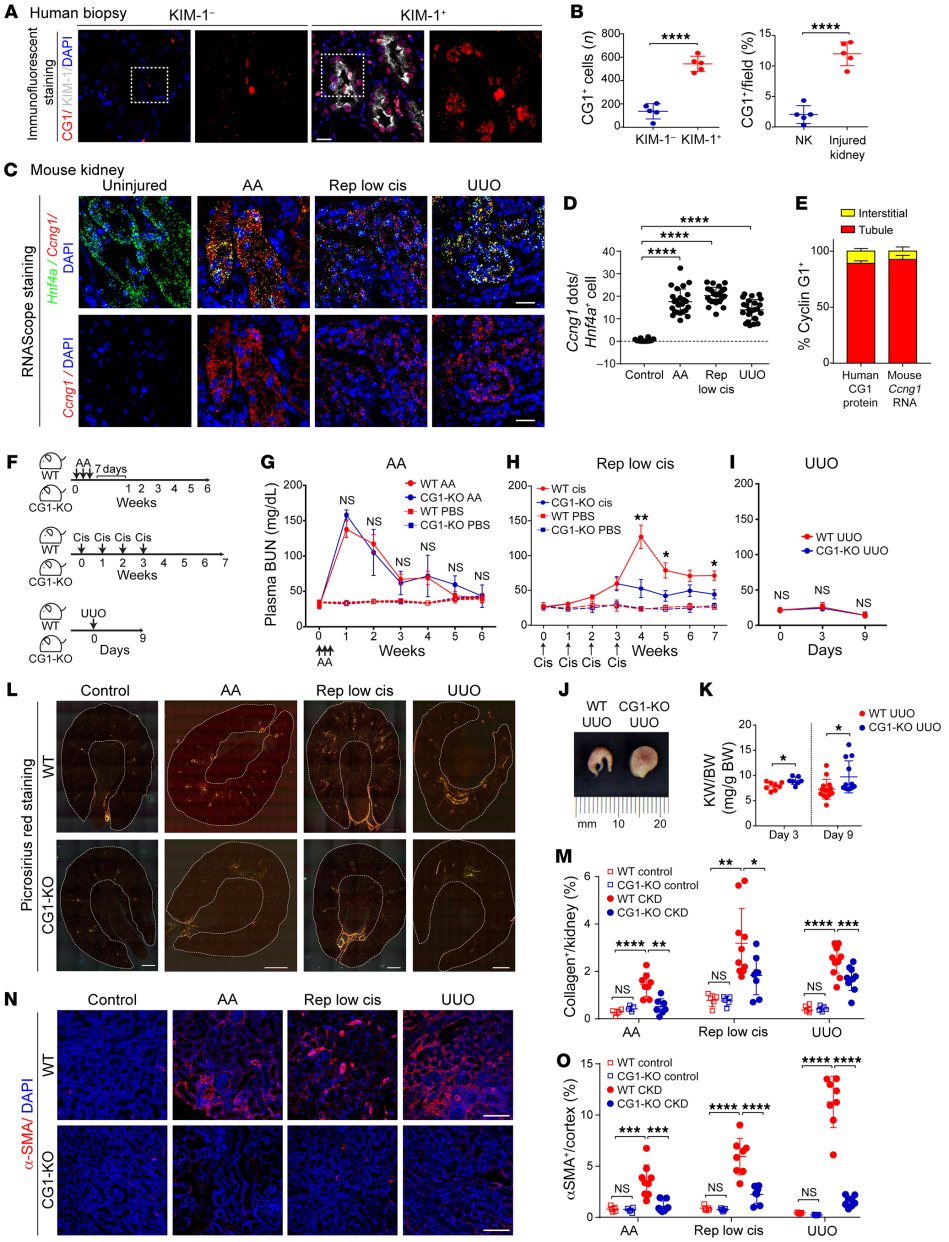
PTC CG1 promotes renal fibrosis in CKD. (**A**) CG1 expression in kidney tissue from patients with and without kidney injury. Scale bar: 20 μm. (**B**) Quantification of CG1 staining of patients in **A**. NK, normal kidney. (**C**) Representative images of RNAScope for *Ccng1* and *Hnf4a* in PTCs of control kidney and CKD models. Scale bars: 20 μm. (**D**) Quantification of number of *Ccng1* RNA dots in *Hnf4a*^+^ PTCs. *n* = 10 in control kidney; *n* = 25 in CKD models. (**E**) Quantification of the percentage of CG1^+^ cells in kidney tubules and all other cell types. (**F**) Schematic diagrams of experimental CKD models with WT and CG1-KO mice. (**G**) Plasma BUN at weekly time points after administration of AA (5 mg/kg every other day for a week). (**H**) Plasma BUN at weekly time points after repeated injection with low-dose cisplatin (5 mg/kg once a week for 4 weeks). (**I**) Plasma BUN on days 0, 3, and 9 after UUO surgery. (**J**) Representative images of obstructed kidneys in WT and CG1-KO mice in the UUO model. (**K**) Ratio of obstructed kidney weight (KW)/body weight. WT UUO (*n* = 14) and CG1-KO UUO (*n* = 12). (**L**) Representative large, scanned images of picrosirius red–stained kidney sections with polarized-light microscopy. Dashed outline indicates region of interest quantified. Scale bars: 500 μm. (**M**) The quantification of collagen deposition area/kidney (%) from **L**. Control (*n* = 5), CKD (*n* = 8–10). (**N**) Representative images of stained kidney for α-SMA. Scale bars: 100 μm. (**O**) Quantitative analysis of α-SMA^+^ area/cortex (%). Control (*n* = 5), CKD (*n* = 8–10). Data are presented as the mean ± SD. *P < 0.05; **P < 0.01; ***P < 0.001; ****P < 0.0001 by unpaired, 2-tailed Student’s *t* test (**B**, **G**, **H**, **I**, and **K**) or 1-way ANOVA with Tukey’s post hoc test (**M** and **O**).

To determine the expression of CG1 in CKD, we stained human kidney samples from patients with or without renal fibrosis for CG1 and KIM-1. CG1 was upregulated in kidneys of patients with CKD and approximately 80% of KIM-1^+^ tubules expressed CG1 (Figure 1, A and B, and Supplemental Figure 1, A and B; supplemental material available online with this article; https://doi.org/10.1172/JCI158096DS1). In mouse kidney tissue, RNAScope analysis revealed that CG1 (*Ccng1*) RNA was upregulated in PTCs (labeled by hepatocyte nuclear factor 4α [*Hnf4a*]; refs. 25, 26), in 3 models of CKD (aristolochic acid [AA], repeated low-dose cisplatin [Rep low cis], and unilateral ureteral obstruction [UUO]) when compared with control uninjured kidneys (Figure 1, C and D). In both mouse and human tissue, approximately 90% of CG1^+^ cells were kidney tubular cells, predominantly KIM-1^+^ PTCs (Figure 1E). CG1 was also upregulated with injury in human kidney organoid cultures (Supplemental Figure 1C). Real-time PCR analysis revealed that CG1 was upregulated on days 28 and 42 after AA injury, while another cyclin G family member, cyclin G2 (*Ccng2*), was not upregulated (Supplemental Figure 1D). These data suggest CG1 is upregulated, predominantly in PTCs, in patients with CKD and CKD animal models.

To investigate the role of CG1 in the AKI-to-CKD transition, we subjected wild-type (WT) and CG1-KO mice to 3 CKD models, AA, Rep low cis, and UUO, as shown in Figure 1F. CG1-KO mice have no developmental or kidney morphological phenotypes (27, 28). Renal function, as assessed by blood urea nitrogen (BUN), was similar in the acute phase of injury for both WT and CG1-KO mice in AA and Rep low cis models (Figure 1, G and H). BUN was unchanged in UUO, due to the presence of an uninjured contralateral kidney (Figure 1I). While BUN returned to baseline in both WT and CG1-KO mice following AA, Rep low cis resulted in prolonged elevated BUN in WT mice, which was reduced in CG1-KO (Figure 1, G and H). At the macroscopic level, kidney structure was preserved in CG1-KO mice when compared with WT mice in the UUO model (Figure 1J), which was supported by a higher kidney weight/body weight ratio (mg/g) in CG1-KO than in WT mice on day 3 and day 9 after UUO surgery (Figure 1K). Picrosirius red staining imaged with polarized-light microscopy (to visualize collagen fibers) demonstrated increased collagen content in injured WT and CG1-KO mice when compared with control mice, but the increase was significantly less in CG1-KO mice (Figure 1, L and M). All 3 injury models induced a significant increase in α-SMA^+^ area in WT kidneys when compared with control, but not in CG1-KO kidneys (Figure 1, N and O). Injured CG1-KO mice also demonstrated reduced kidney injury markers, KIM-1 and cleaved caspase 3, and preserved Na^+^/K^+^-ATPase staining in the chronic phase of injury compared with injured WT mice (Supplemental Figure 2, A–D). Expression of profibrotic factors *Ccn2* (CTGF), *Timp2*, *Pdgfrb*, and *Col1a1* was increased at the mRNA level in WT kidneys but not in injured CG1-KO kidneys (Supplemental Figure 2, E and F). Taken together, these data demonstrate that CG1 regulates fibrosis progression in CKD.

### Induction of G_2_/M arrest in CG1-KO mice only partially restores fibrosis.

**Figure 2:**
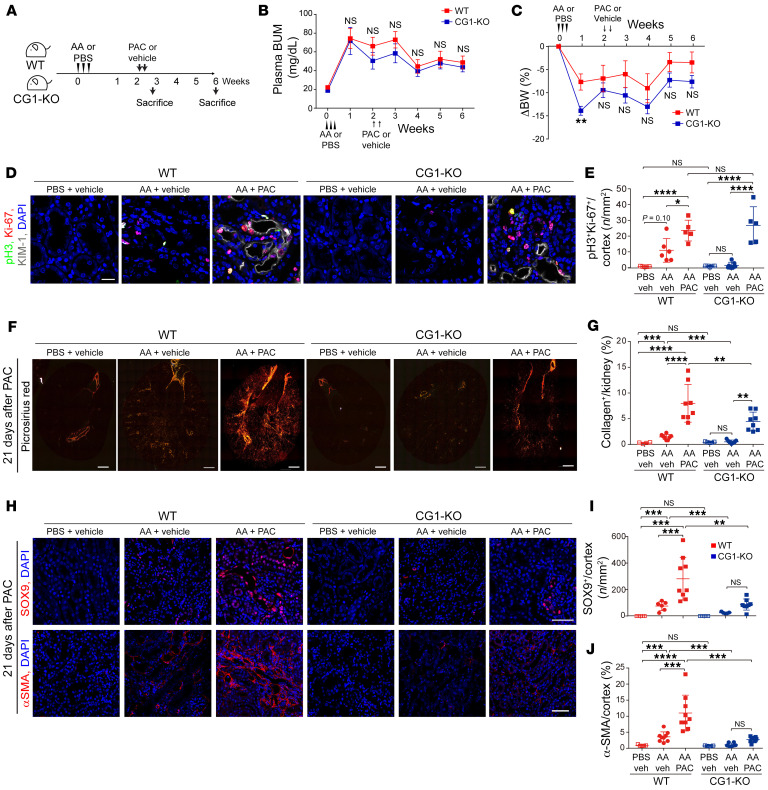
Inducing G_2_/M arrest in CG1-KO mice does not reverse the protective phenotype. (**A**) A schematic diagram of injection with AA (5 mg/kg) and paclitaxel (PAC) (20 mg/kg). (**B**) Plasma BUN level over time and (**C**) body weight (compared to day 0) in WT (*n* = 9) and CG1-KO mice (*n* = 8–10). (**D**) Representative images of p-H3^+^Ki-67^+^ PTCs from kidneys of WT and CG1-KO mice on day 42 following AA administration. Scale bar: 20 μm. (**E**) Quantification of the number of p-H3^+^Ki-67^+^ PTCs in cortex (*n*/mm^2^). (**F**) Representative images of picrosirius red–stained kidney sections from mice treated with PBS (*n* = 5), AA (*n* = 8), AA + PAC (*n* = 8), and (**G**) the corresponding quantification of collagen deposition area/cortex (%). Scale bars: 500 μm. (**H**) Representative images of the kidneys stained for SOX9 (upper) and α-SMA (lower). Scale bars: 50 μm. (**I** and **J**) The corresponding quantification of the number of SOX9^+^ PTCs/cortex (*n*/mm^2^), and α-SMA^+^ area/cortex (%) in left panel. PBS (*n* = 5) and AA + PAC (*n* = 8–9). Data are presented as the mean ± SD. *P < 0.05; **P < 0.01; ***P < 0.001; ****P < 0.0001 by unpaired, 2-tailed Student’s *t* test (**B** and **C**) or 1-way ANOVA with Tukey’s post hoc test (**E**, **G**, **I**, and **J**).

We hypothesized that CG1 induced fibrosis by promoting G_2_/M arrest, and activation of G_2_/M arrest in CG1-KO mice would reverse the protective phenotype and promote fibrosis. To test this hypothesis, we utilized paclitaxel (PAC), which we have previously shown to induce G_2_/M arrest and fibrosis in injured but not uninjured kidneys, to promote G_2_/M arrest in AA-treated WT and CG1-KO mice (10). Injection of PAC 2 weeks after AA administration raised plasma BUN to similar levels in WT and CG1-KO mice (Figure 2, A and B). The reduction in body weight (compared with day 0) was also similar between WT and CG1-KO mice throughout the experiment except for week 1, where CG1-KO had a greater reduction in body weight (Figure 2C). Without PAC, there were significantly fewer p-H3^+^Ki-67^+^ double-positive G_2_/M-phase cells in CG1-KO mice following AA, Rep low cis, or UUO injury compared with injured WT mice (Figure 2, D and E, and Supplemental Figure 2, G–I). Injection of PAC significantly increased the number of G_2_/M-phase cells in both WT and CG1-KO mice compared with AA alone, and there was no significant difference in the number of G_2_/M-phase cells in WT AA- and PAC-treated compared to CG1-KO AA- and PAC-treated kidneys (Figure 2, D and E). Acute tubular injury, assessed by KIM-1 expression, was also similar between WT and CG1-KO mice following PAC injection (Supplemental Figure 3, A–C). However, picrosirius red staining revealed kidney fibrosis was significantly reduced in CG1-KO AA + PAC mice compared with WT AA + PAC mice (Figure 2, F and G). Chronic phase (day 42) KIM-1^+^ staining was attenuated in CG1-KO AA + PAC mice, but not WT AA + PAC mice (Supplemental Figure 3, D and E). Immunofluorescent staining revealed SOX9 and α-SMA were increased in WT mice treated with AA + PAC but no significant increase was observed in CG1-KO mice (Figure 2, H–J). Taken together, these data suggest that the profibrotic actions of CG1 are only partially dependent on G_2_/M arrest.

### Inducing G_2_/M arrest in vitro does not induce fibrotic or dedifferentiation markers in primary PTCs.

**Figure 3:**
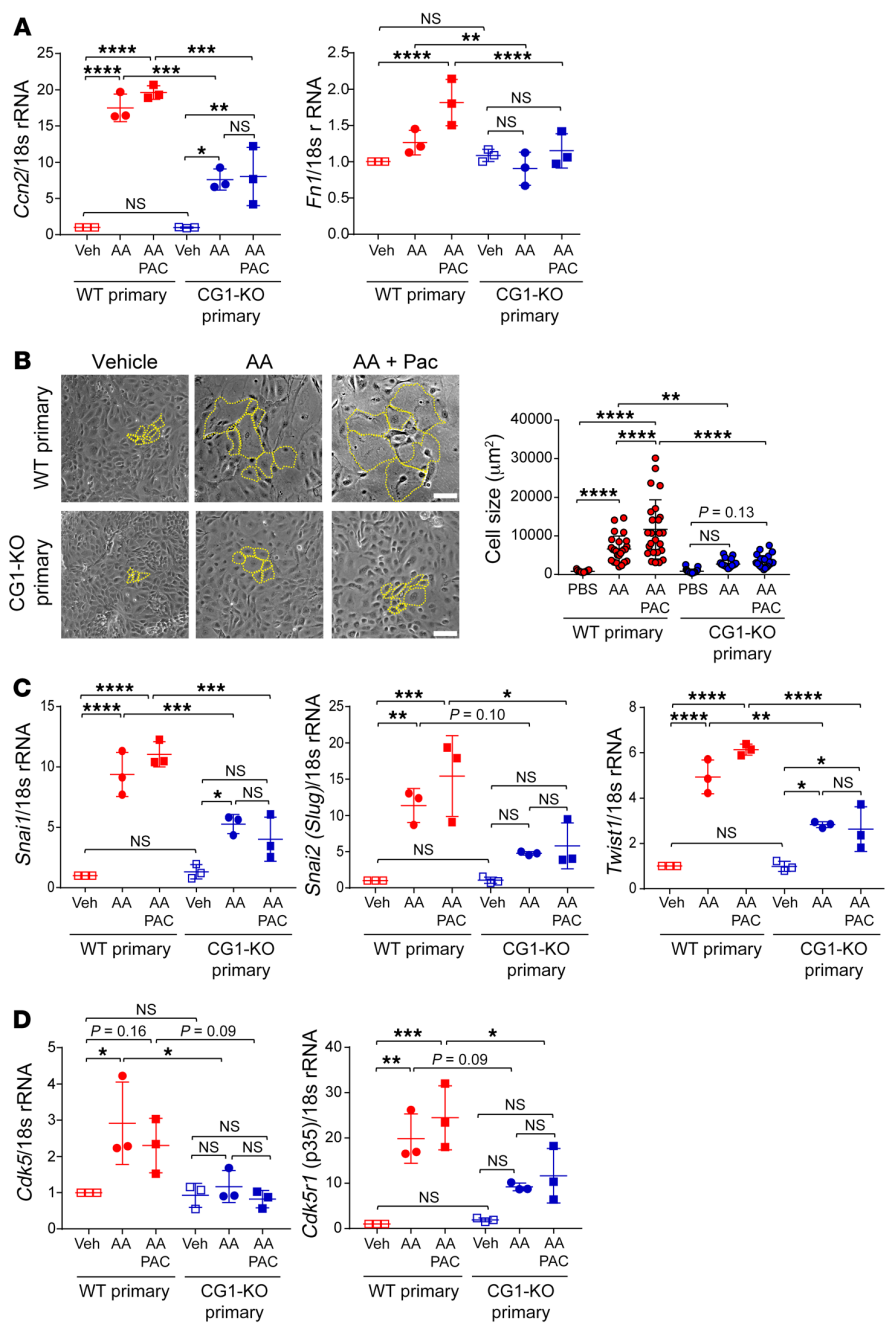
Inducing G_2_/M arrest in vitro does not induce fibrotic or dedifferentiation markers in primary PTCs. (**A**) Real-time PCR analysis of *Ctgf* and *Fn1* in WT and CG1-KO primary PTCs treated with PBS, AA, or AA + PAC for 7 days. *n* = 3 independent experiments. (**B**) Representative phase-contrast images of WT and CG1-KO primary PTCs treated with PBS, AA, or AA + PAC for 7 days, and the quantification of individual cell size (μm^2^) of WT and CG1-KO primary PTCs in PBS, AA, and AA + PAC. *n* = 25 each. A portion of cells are outlined in yellow to demonstrate cell size; all cells/field were quantified. Scale bars: 100 μm. (**C**) Dedifferentiation marker mRNA expression in WT and CG1-KO primary PTCs treated with PBS, AA, or AA + PAC for 7 days. *n* = 3 independent experiments. (**D**) Real-time PCR analysis of *Cdk5* and *Cdk5r1* (p35) in WT and CG1-KO primary PTCs treated with PBS, AA, or AA + PAC for 7 days. *n* = 3 independent experiments. AA, 5 μg/mL; PAC, 1 μM. Data are presented as the mean ± SD. *P < 0.05; **P < 0.01; ***P < 0.001; ****P < 0.0001 by 1-way ANOVA with Tukey’s post hoc test.

To confirm whether G_2_/M arrest can induce profibrotic responses in CG1-KO PTCs, we analyzed primary PTCs from WT and CG1-KO mice treated with AA, which we have previously shown to induce a profibrotic phenotype in vitro (10), or AA and PAC. PAC treatment induced p-H3 in most cells in vitro (Supplemental Figure 3F). Real-time PCR analysis revealed *Ccn2* (CTGF) and *Fn1* mRNA were induced in WT PTCs with a combination of AA and PAC, but the addition of PAC did not upregulate either factor in CG1-KO primary PTCs (Figure 3A). We observed that AA treatment alone induced an increase in cell size and the combination of AA and PAC further increased cell size in WT PTCs; however, neither AA nor AA + PAC treatment increased cell size in CG1-KO primary PTCs (Figure 3B). Treatment with AA or AA + PAC led to an increase in the dedifferentiation-related genes *Snail*, *Slug*, and *Twist* in WT primary PTCs, all of which were reduced in CG1-KO primary cells compared with WT (Figure 3C). Both AA and AA + PAC treatment promoted the expression of CDK5 (a downstream target of CG1) and its activating binding partner p35 in WT PTCs, while there was no significant increase in CG1-KO primary PTCs (Figure 3D). No difference in growth rate following scratch wound assay was observed between WT and CG1-KO PTCs (Supplemental Figure 3, G and H). Taken together, these data demonstrate that PAC does not induce dedifferentiation or profibrotic cytokine production compared to AA alone, suggesting G_2_/M arrest does not influence dedifferentiation. CG1-KO cells have reduced upregulation of profibrotic and dedifferentiation markers after injury, suggesting CG1 may regulate dedifferentiation and fibrosis pathways in PTCs independently of G_2_/M arrest.

### CG1 promotes PTC dedifferentiation in CKD but not AKI.

**Figure 4:**
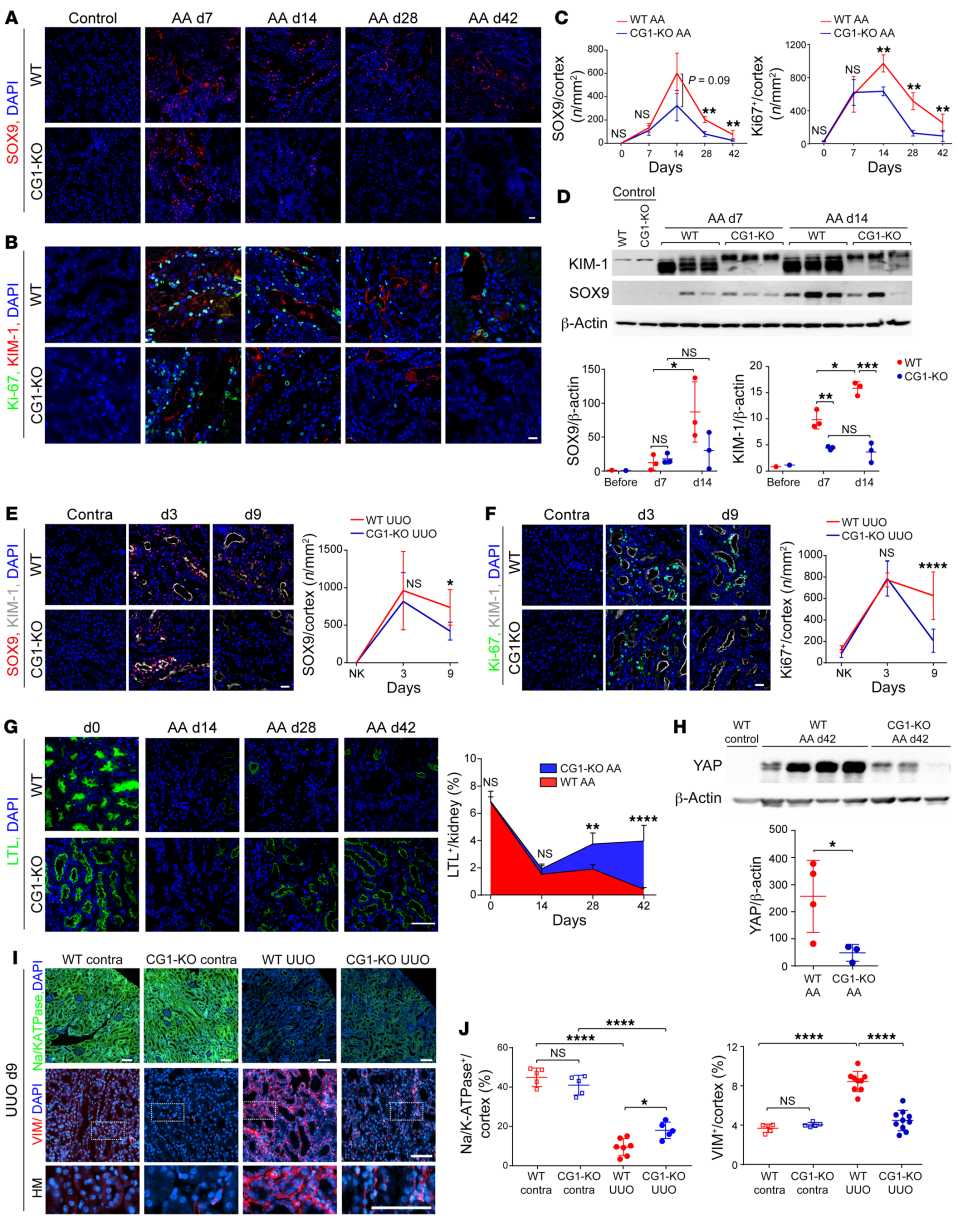
CG1 modulates PTC dedifferentiation and proliferation in CKD. (**A** and **B**) Representative images of SOX9-labeled kidney sections from WT and CG1-KO mice in acute (day 7) and chronic (day 42) phases of AA. Scale bar: 20 μm (**C**) Corresponding quantification of the number of SOX9^+^ cells/kidney. Control (*n* = 5) and injured kidney (*n* = 3–6). (**D**) Western blot analysis of KIM-1, SOX9, and β-actin in acute phase of AA, including day 7 and day 14 after administration of AA and the corresponding quantification of SOX9/β-actin and KIM-1/β-actin. (**E** and **F**) Representative images of SOX9- and Ki-67–labeled kidney sections from WT and CG1-KO mice on day 3 and day 9 of UUO and corresponding quantification of the number of SOX9^+^ or Ki-67^+^ cells/kidney. Control (NK; *n* = 5) and injured kidney (*n* = 5–9). Scale bar: 20 μm. (**G**) Representative images of LTL staining in kidneys on days 0, 14, 28, and 42 following AA and corresponding quantification. Scale bar: 50 μm. (**H**) Western blotting analysis of YAP in whole-kidney lysates of WT and CG1-KO mice following AA. (**I**) Representative images of Na^+^/K^+^-ATPase– or VIM-stained kidney sections following UUO. Scale bars: 100 μm. HM, high magnification. (**J**) The corresponding quantification of Na^+^/K^+^-ATPase^+^ or VIM^+^ area/cortex (%). Control (*n* = 4–6) and injured kidney (*n* = 8–9). Scale bar: 100 μm. Data are presented as the mean ± SD. *P < 0.05; **P < 0.01; ***P < 0.001; ****P < 0.0001 by 1-way ANOVA with Tukey’s post hoc test.

To test whether CG1 modulates dedifferentiation after kidney injury, we stained WT and CG1-KO kidneys for dedifferentiation and proliferation markers at different time points following injury. Immunofluorescent staining revealed many dedifferentiated SOX9^+^ PTCs and proliferative Ki-67^+^ PTCs were observed in the acute phase (7 days) of AA in both WT and CG1-KO mice, and no statistical difference was detected (Figure 4, A–C). However, the number of SOX9^+^ or Ki-67^+^ PTCs remained elevated in WT mice during the chronic phase (days 14–42), while CG1-KO mice showed a significant decrease in the number of SOX9^+^ or Ki-67^+^ PTCs at these later time points (Figure 4, A–C). Western blot analysis from kidney cortical lysates confirmed SOX9 was increased in both WT and CG1-KO mice on day 7 following administration of AA; however, on day 14, SOX9 expression continued to increase in WT mice while remaining stable in the CG1-KO mice (Figure 4D). A similar trend for SOX9 and Ki-67 expression was observed in the UUO and Rep low cis models (Figure 4, E and F, and Supplemental Figure 4, A–E). These data suggest that KO of CG1 does not alter the dedifferentiation response in the early recovery phase following AKI.

To determine whether CG1 regulates dedifferentiation in the chronic phase of injury, we examined the expression of differentiation and dedifferentiation markers in chronically injured WT and CG1-KO kidneys (day 42 AA, day 49 Rep low cis, and day 9 UUO). *Lotus* *tetragonolobus* lectin–positive (LTL^+^) area (identifying intact PTC brush border) was decreased on day 14 after AA injection in both WT and CG1-KO kidneys and recovered on days 28 and 42 in CG1-KO, but not WT, kidneys (Figure 4G). We found that YAP, a known regulator of dedifferentiation in many organs (29, 30), was upregulated 42 days after AA injection in WT mice but not CG1-KO mice (Figure 4H and Supplemental Figure 4F). The differentiation marker Na^+^/K^+^-ATPase was downregulated in both WT and CG1-KO injured kidneys, but the injured CG1-KO had significantly more positive area compared with injured WT (Figure 4, I and J). Vimentin (VIM) increased in WT but not CG1-KO mice after injury (Figure 4, I and J). Expression of PTC dedifferentiation markers *Prom1* (CD133), *Acta2* (α-SMA), *VIM*, *Pax2*, and *Pax8* (6, 7, 31–36) was significantly reduced in CG1-KO compared with WT in the chronic injury phase of AA, Rep low cis, and UUO (Supplemental Figure 4G). No difference in p53 (a known regulator of CG1) levels was seen between WT and CG1-KO mice after injury (Supplemental Figure 4H). The senescence marker p21 was upregulated in WT primary PTCs but not CG1-KO following AA exposure (Supplemental Figure 4I). Taken together, these data suggest that CG1 does not play a role in the adaptive dedifferentiation and repair following AKI but may promote the maladaptive dedifferentiation of PTCs seen in CKD.

### CG1 promotes PTC dedifferentiation through the activation of CDK5.

**Figure 5:**
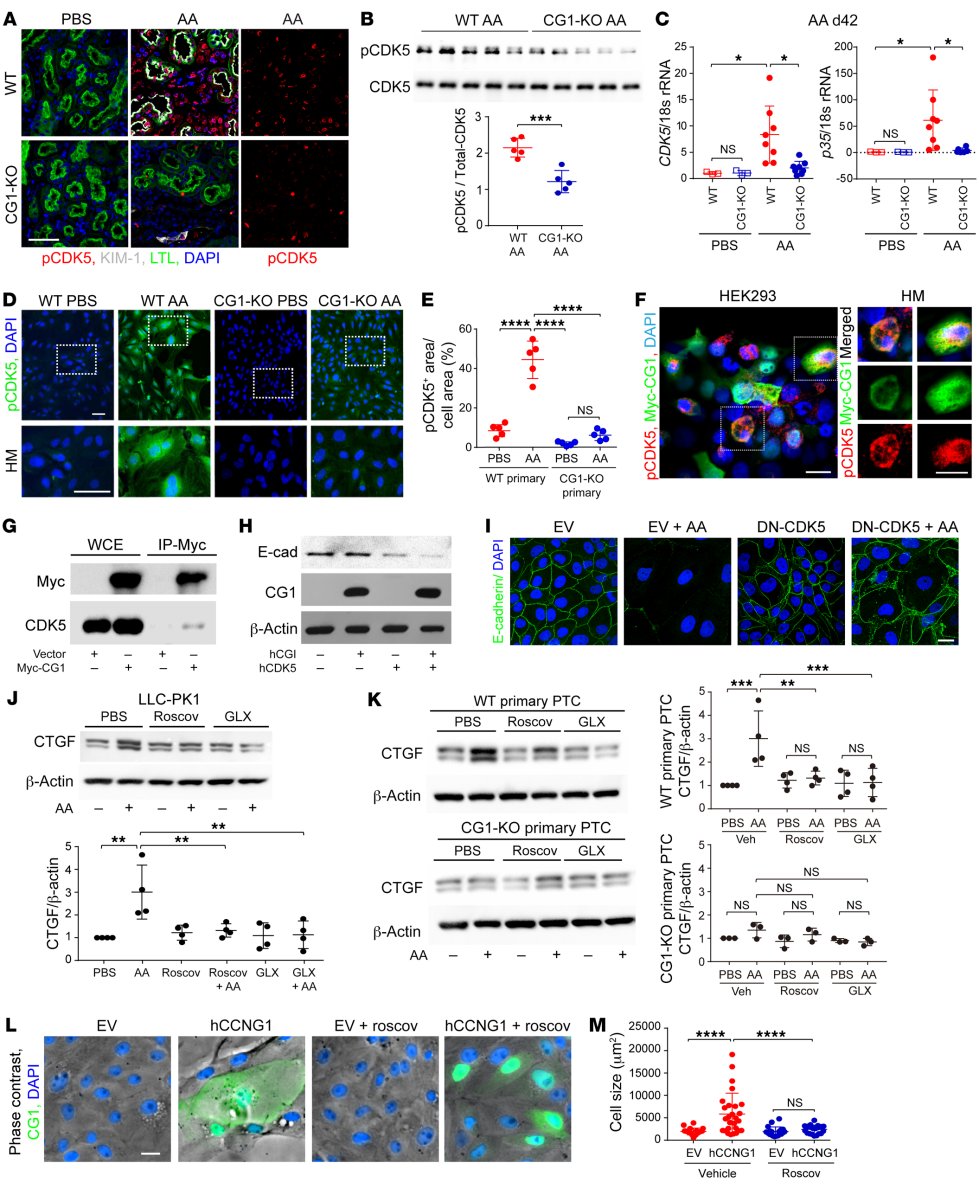
CG1 binds to and activates CDK5 in PTCs. (**A**) Representative images of p-CDK5–labeled kidneys from day 42 WT PBS, CG1-KO PBS, WT AA, and CG1-KO AA. Scale bar: 50 μm. (**B**) Western blot analysis of p-CDK5 in AA-treated WT and CG1-KO kidneys and quantitative analysis in WT AA (*n* = 5) and CG1-KO AA (*n* = 5). (**C**) Real-time PCR analysis of CDK5 and p35 following PBS or AA treatment (PBS, *n* = 3; AA, *n* = 8). (**D** and **E**) Representative images and quantification of p-CDK5 in WT and CG1-KO primary PTCs treated with PBS or AA (5 μg/mL) for 7 days. Scale bars: 50 μm. *n* = 5 in each group. HM, high magnification. (**F**) Representative images of p-CDK5 in hCG1-overexpressing HEK293T cells. Scale bars: 20 μm. (**G**) Representative Western blots of CG1-Myc and CDK5 in whole-cell extract (WCE) of Myc-CG1–overexpressing HEK293T and immunoprecipitation by Myc. (**H**) Western blot analysis of E-cadherin, CG1, and total CDK5 in LLC-PK1 cells transfected with human CG1 (hCG1) and/or hCDK5 for 48 hours. (**I**) Representative images of E-cadherin in AA-treated LLC-PK1 cells transfected with DN-CDK5 or empty vector (EV). Scale bar: 20 μm. (**J**) Representative Western blot analysis of CTGF in LLC-PK1 cells treated with/without AA (2.5 μg/mL) and PBS, roscovitine, or RO5454291 from Glixx labs (GLX). (**K**) Western blot analysis of CTGF in WT and CG1-KO primary PTCs treated with AA (5 μg/mL) for 7 days with/without CDK5 inhibitors roscovitine (8 μM) and GLX (50 μM). (**L** and **M**) Representative phase-contrast images and quantification of cell size in human CG1–overexpressing (hCCNG1-overexpressing) LLC-PK1 cells with/without roscovitine. Scale bar: 20 μm. *n* = 25 in each. Data are presented as the mean ± SD. *P < 0.05; **P < 0.01; ***P < 0.001; ****P < 0.0001 by unpaired, 2-tailed Student’s *t* test (**B**) or 1-way ANOVA with Tukey’s post hoc test (**C**, **E**, **J**, **K**, and **M**).

To investigate how CG1 regulates dedifferentiation, we analyzed the activation of CDK5 through its phosphorylation at tyrosine 15. Immunofluorescent staining demonstrated increased p-CDK5 in the nuclei of PTCs in WT kidneys following AA treatment, while staining was minimal in CG1-KO kidneys (Figure 5A). Similarly, Western blot analysis revealed p-CDK5 expression was reduced in CG1-KO mice compared with WT mice 42 days following AA injection (Figure 5B). Expression of both *Cdk5* and its activator *Cdk5r1* (p35) were increased at the mRNA level in WT CKD but remained near baseline in CG1-KO kidneys in all 3 CKD models tested (Figure 5C and Supplemental Figure 5, A and B). Treatment of primary PTCs with AA upregulated p-CDK5 in WT cells, but no significant increase was observed in CG1-KO cells (Figure 5, D and E, and Supplemental Figure 5C). CDK5-GFP translocated to the nucleus following AA exposure and induced CTGF expression (Supplemental Figure 5, D and E). Transfection of CG1 in human embryonic kidney 293T (HEK293T) cells resulted in increased p-CDK5 (Figure 5F). To determine whether CG1 interacts with CDK5, HEK293T were transfected with Myc-CG1 and the lysates were immunoprecipitated (IP) using antibodies directed against Myc. Western blot analysis of the precipitate confirmed the binding of CG1 to CDK5 in PTCs (Figure 5G). Thus, CG1 interacts with CDK5, and CG1 regulates CDK5 phosphorylation/activation in PTCs.

Next, we examined whether CDK5 regulates the expression of differentiation and dedifferentiation markers in vitro. E-cadherin protein expression decreased in cells overexpressing CDK5, which was further decreased by the combination of CDK5 and CG1, similar to AA treatment (Figure 5H and Supplemental Figure 5F). Inhibition of CDK5 through transfection of dominant-negative CDK5 (DN-CDK5) prevented the reduction in E-cadherin expression (Figure 5I). To investigate the therapeutic potential of CDK5 inhibition we utilized CDK5 inhibitors roscovitine (Roscov), which preferentially inhibits CDK5 by competitively binding to the ATP-binding site (37), and RO5454291 from Glixx labs (GLX), which inhibits CDK5 activation by disrupting the interaction of p25 and CDK5 (38). Inhibition of CDK5 with Roscov or GLX prevented AA-induced upregulation of CTGF and VIM, while preventing the downregulation of E-cadherin, in LLC-PK1 cells but had no effect on E-cadherin levels in CG1-KO primary cells (Figure 5J and Supplemental Figure 5, G–J). Inhibition of CDK5 prevented AA-induced CTGF upregulation in WT primary PTCs but had no effect on CG1-KO PTCs (Figure 5K). CDK5 inhibition also prevented changes in cell morphology and β-galactosidase activity induced by CG1 transfection or AA treatment in LLC-PK1 cells (Figure 5, L and M, and Supplemental Figure 6, A–D). The upregulation of profibrotic markers or dedifferentiation markers (*Tgfb1*, *Pdgfa*, *Pdgfr*, *Timp2*, *Acta2*, *Sox9*, *Vim*, and *Pax2*) was significantly reduced at the mRNA level by GLX treatment (Supplemental Figure 6, E and F). CDK5 inhibition did not significantly alter the expression of dedifferentiation markers *Prom1* (CD133), *Cd24*, or *Vim* in CG1-KO primary cells (Supplemental Figure 6, G and H). These data suggest that CDK5 is associated with PTC dedifferentiation and fibrosis marker expression in injured PTCs.

### Inhibition of CDK5 reduces renal fibrosis and dedifferentiation in CKD.

**Figure 6:**
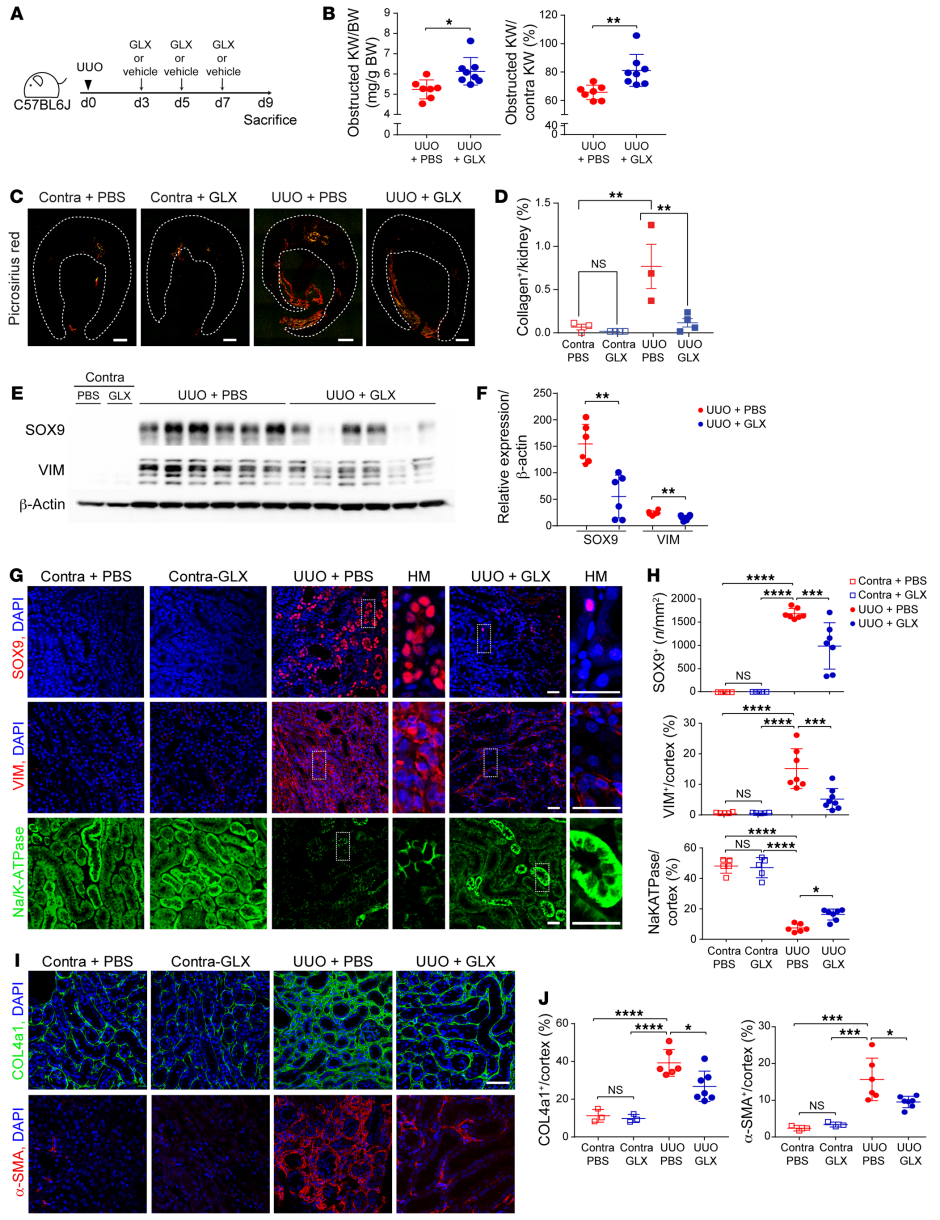
CG1/CDK5 axis regulates PTC dedifferentiation and fibrosis in CKD rodents. (**A**) Schematic diagram of the UUO model with CDK5 inhibitor GLX. (**B**) Quantitative analysis of obstructed kidney weight (KW)/body weight (mg/g BW) and obstructed/contralateral kidneys (%) following UUO with GLX or vehicle. (**C**) Large, scanned images of picrosirius red–stained kidneys imaged by polarized-light microscopy from UUO plus GLX or vehicle. Dashed outline indicates region of interest quantified. Scale bars: 500 μm. (**D**) Quantification of picrosirius red^+^ area/cortex (%) in **C**. Contralateral kidney of vehicle, *n* = 5; contralateral kidney of GLX, *n* = 5; UUO with vehicle, *n* = 6; UUO with GLX, *n* = 6. (**E**) Western blot analysis of SOX9 and VIM in whole-kidney lysates of UUO and contralateral kidneys with vehicle or GLX, and (**F**) quantification of SOX9/β-actin and VIM/β-actin. (**G**) Representative images of dedifferentiation markers (SOX9, Na^+^/K^+^-ATPase, and VIM) in kidneys from contralateral or UUO kidneys treated with vehicle or GLX. Scale bars: 25 μm. HM, high magnification. (**H**) Quantitative analysis of the number of SOX9^+^ PTCs/cortex (*n*/mm^2^), Na^+^/K^+^-ATPase^+^/cortex (%), or VIM^+^ area/cortex (%) in contralateral or UUO kidney treated with vehicle or GLX. Contralateral kidney of vehicle, *n* = 5; contralateral kidney of GLX, *n* = 5; UUO with vehicle, *n* = 7; UUO with GLX, *n* = 7–8. (**I**) Representative images of collagen type IV α1 chain (COL4a1) in contralateral or UUO kidney with vehicle or GLX. Scale bar: 50 μm. (**J**) The corresponding analysis of COL4a1^+^ area/cortex (%). Contralateral kidney of vehicle, *n* = 3; contralateral kidney of GLX, *n* = 3; UUO with vehicle, *n* = 6; UUO with GLX, *n* = 7. Data are presented as the mean ± SD. *P < 0.05; **P < 0.01; ***P < 0.001; ****P < 0.0001 by unpaired, 2-tailed Student’s *t* test (**B**) or 1-way ANOVA with Tukey’s post hoc test (**D**, **F**, **H**, and **J**).

To determine whether pharmacological inhibition of CDK5 ameliorates fibrosis in CKD, we induced UUO in WT mice and treated with vehicle or GLX starting on day 3 after surgery (Figure 6A). We analyzed the obstructed kidney weight/body weight and the ratio of the obstructed kidney weight/contralateral kidney weight and found that both increased in the GLX treatment group compared with vehicle (Figure 6B). Picrosirius red staining revealed GLX injection inhibited kidney fibrosis (Figure 6, C and D). KIM-1^+^ staining was attenuated by administration of GLX compared with vehicle (Supplemental Figure 7, A and B). Western blotting demonstrated an upregulation of SOX9 and VIM in UUO, which was reduced by treatment with GLX (Figure 6, E and F). Similarly, immunofluorescent staining demonstrated that the increase in SOX9^+^ PTCs and VIM^+^ area in UUO kidneys treated with vehicle were reduced by GLX treatment (Figure 6, G and H). In contrast, Na^+^/K^+^-ATPase^+^ area was preserved by GLX treatment in UUO kidneys compared with vehicle treatment (Figure 6, G and H). The upregulation of collagen type IV (indicative of increased extracellular matrix production) and α-SMA observed in PBS-treated mice following UUO was reduced by administration of GLX (Figure 6, I and J). These data suggest that inhibition of CDK5 prevents dedifferentiation and renal fibrosis.

### Tubule-specific deletion of CDK5 inhibits renal fibrosis and dedifferentiation in CKD.

**Figure 7:**
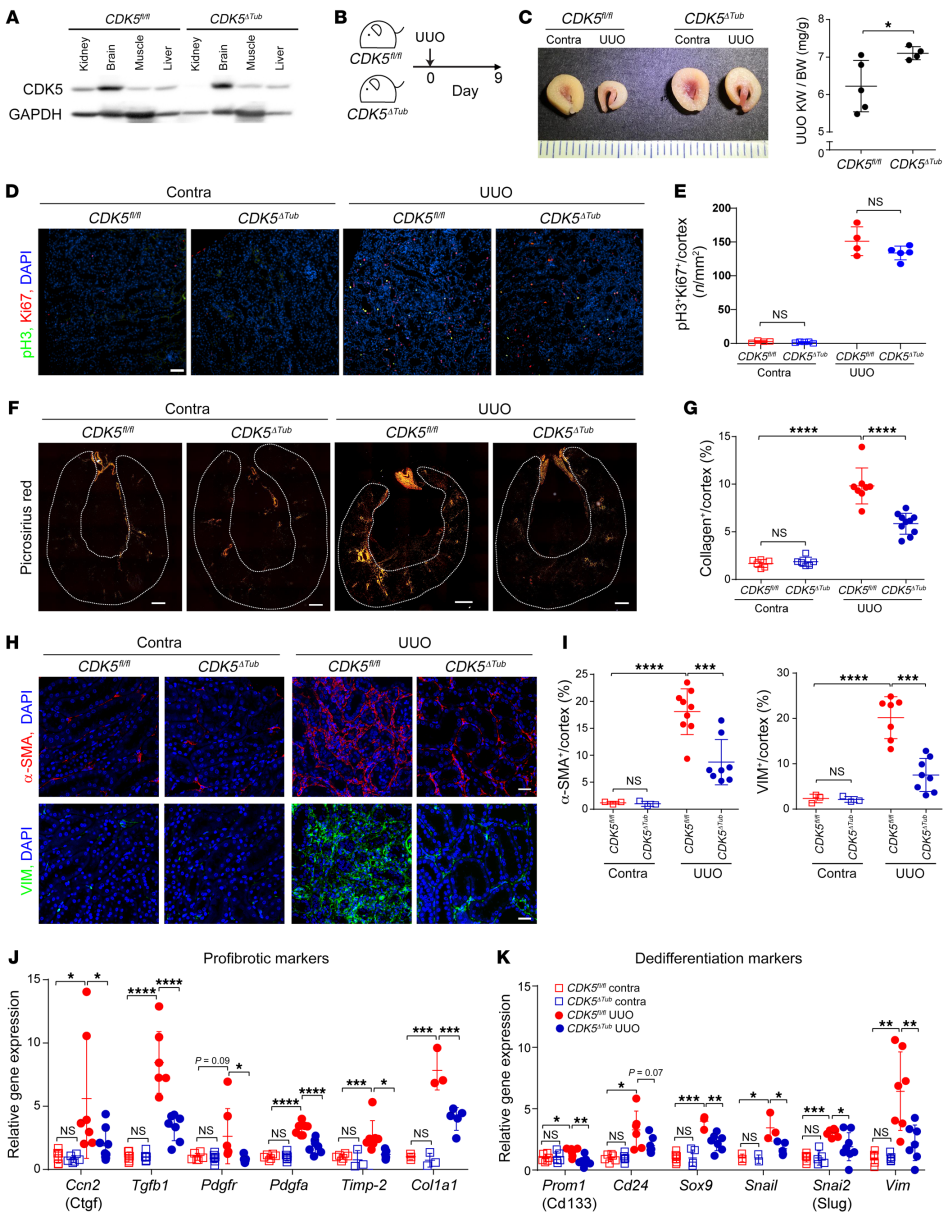
Tubule-specific deletion of *CDK5* inhibits dedifferentiation and fibrosis. (**A**) Western blot analysis of CDK5 levels in *CDK5^fl/fl^* and *CDK5*^ΔTub^ mice in the kidney and different organs. (**B**) Schematic representation of the UUO model. (**C**) Representative images of thick kidney slices from *CDK5^fl/fl^* and *CDK5*^ΔTub^ and quantification of kidney weight (KW)/body weight following UUO. *n* = 4–5 kidneys. Scale: 1 mm/tick. (**D** and **E**) Representative images of p-H3 and Ki-67 staining in *CDK5^fl/fl^* and *CDK5*^ΔTub^ kidneys following UUO and quantification of the staining. Scale bar: 50 μm. (**F** and **G**) Representative polarized-light images of picrosirius red–stained *CDK5^fl/fl^* and *CDK5*^ΔTub^ kidneys and quantification of the positive signal. *n* = 8–9 independent experiments. Dashed outline indicates region of interest quantified. Scale bars: 500 μm. (**H** and **I**) Representative immunofluorescence images of α-SMA– and vimentin-stained *CDK5^fl/fl^* and *CDK5*^ΔTub^ kidneys following UUO and quantification of the staining. Scale bars: 20 μm. (**J** and **K**) Real-time PCR analysis of profibrotic and dedifferentiation markers in *CDK5^fl/fl^* and *CDK5*^ΔTub^ kidneys. *n* = 3–6 animals. Data are presented as the mean ± SD. *P < 0.05; **P < 0.01; ***P < 0.001; ****P < 0.0001 by unpaired, 2-tailed Student’s *t* test (**C**) or 1-way ANOVA with Tukey’s post hoc test (**E**, **G**, and **I**–**K**).

To confirm that tubular epithelial cell expression of CDK5 contributes to injury, we bred *CDK5^fl/fl^* mice with *Six2*-*Cre* mice to generate mice lacking CDK5 in kidney epithelial cells (*CDK5*^ΔTub^ mice; podocytes proximal and distal tubule structures up to the connection with the collecting duct; ref. 39). *CDK5*^ΔTub^ mice have reduced expression of CDK5 in kidney lysates, but not in lysates of other organs (Figure 7A). *CDK5*^ΔTub^ mice maintained a higher kidney weight–to–body weight ratio than control mice following UUO, and no difference was observed in contralateral kidneys (Figure 7, B and C, and Supplemental Figure 7C). Immunostaining demonstrated that both WT and *CDK5*^ΔTub^ mice had increased p-H3^+^Ki-67^+^ double-positive G_2_/M-phase cells following UUO, and no significant difference was observed (Figure 7, D and E). Picrosirius red staining revealed reduced fibrosis in *CDK5*^ΔTub^ mice compared with control mice following UUO (Figure 7, F and G). Dedifferentiation markers α-SMA and VIM were increased after UUO in both *CDK5*^ΔTub^ and control mice, but the increase was significantly lower in *CDK5*^ΔTub^ mice (Figure 7, H and I). Real-time PCR analysis demonstrated a significant reduction in profibrotic and dedifferentiation markers in *CDK5*^ΔTub^ mice compared with controls after UUO (Figure 7, J and K). KIM-1^+^ staining was reduced in *CDK5*^ΔTub^ compared with controls after UUO (Supplemental Figure 7, D and E). Expression of dedifferentiation markers *Cdh6* and *Vcam1* was also reduced in *CDK5*^ΔTub^ compared with *CDK5^fl/fl^* on day 9 after UUO (Supplemental Figure 7F). Thus, while the deletion of *CDK5* in kidneys using *Six2*-*Cre* did not prevent the increase in G_2_/M-phase cells, it did inhibit the induction of dedifferentiation markers and kidney fibrosis.

## Discussion

There are 3 main conclusions from the current study. First, while CG1 regulates G_2_/M arrest, its role in CKD is not dependent on G_2_/M arrest. We found that deletion of CG1 inhibited G_2_/M arrest and fibrosis progression. Pharmacological induction of G_2_/M arrest, however, did not fully reverse the antifibrotic phenotype associated with CG1 KO. Second, CG1 promoted dedifferentiation through the activation of CDK5. CG1 binds to CDK5, promoting its phosphorylation and activation. Inhibition or deletion of CDK5 reduced dedifferentiation and fibrosis progression in chronically injured kidneys. Third, CG1 and CDK5 were not required for repair after AKI but promoted chronic PTC dedifferentiation, which drives CKD progression.

**Figure 8:**
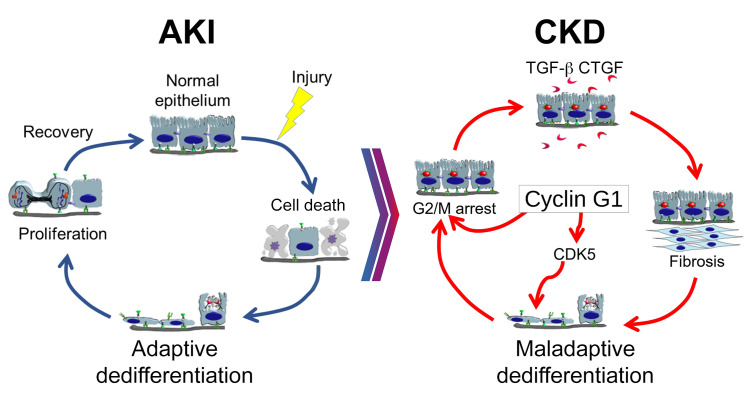
Model of CG1 regulation of dedifferentiation and CKD. Following AKI, there is loss of tubule epithelial cells by cell death followed by adaptive dedifferentiation and proliferation of the surviving cells. Once the surviving cells divide to repair the damaged epithelium, they redifferentiate into a normal epithelium. Long-term expression of CG1 induces maladaptive dedifferentiation, in which the epithelial cells undergo G_2_/M arrest, secretion of profibrotic cytokines, and induction of tubulointerstitial fibrosis. Mechanistically, CG1 expression upregulates and activates CDK5, leading to dedifferentiation. Dedifferentiation precedes the induction of the cell cycle and G_2_/M arrest.

Both G_2_/M arrest and dedifferentiation have been implicated in progressive fibrosis leading to CKD; however, few studies have explored the connection and interplay between these pathways. CG1-KO kidneys have reduced G_2_/M-phase cells following injury; however, our data indicate that induction of G_2_/M arrest in the absence of CG1 leads to greatly reduced profibrotic activity. G_2_/M arrest also does not regulate PTC dedifferentiation in WT or CG1-KO mice or isolated PTCs. This indicates that the profibrotic effects of G_2_/M arrest occur downstream of CG1/CDK5-induced dedifferentiation. This is logical because mature, differentiated PTCs must undergo cellular dedifferentiation before entering the cell cycle and reaching the G_2_/M transition. On the other hand, it is clear that promoting G_2_/M arrest in WT mice, with CG1/CDK5-induced dedifferentiation, dramatically worsens fibrosis progression. These findings suggest that dedifferentiation is not dependent on G_2_/M arrest. The profibrotic effects of G_2_/M arrest, however, are dependent on the cells reaching a dedifferentiated state. In the pathological setting, both dedifferentiation and G_2_/M are likely necessary to create the profibrotic phenotype observed in CKD (Figure 8).

CG1 is known to regulate the cell cycle through binding and activation of CDK5 (17–19). The CG1-CDK5 interaction is not known to promote a pathological response in the kidney or other organs. CDK5 is a key regulator of differentiation status in neurons and is often considered to be a neuron-specific kinase because the expression of CDK5 and its activator p35 are largely limited to terminally differentiated cells under basal conditions (22, 40). Previous studies in the kidney have focused on the role of CDK5 in terminally differentiated podocytes (41–43). Here we demonstrate that CDK5 and p35 are expressed in chronically injured PTCs, and the expression is regulated by CG1. Interestingly, deletion of CDK5 from kidney epithelial cells did not reduce the number of G_2_/M-arrested cells but did lower the number of dedifferentiated cells. This suggests that CG1 regulates dedifferentiation through CDK5 and G_2_/M arrest through a separate mechanism. Thus, CDK5 contributes to the pathobiology of CKD, and targeting the CG1/CDK5 pathway via inhibition of CDK5 is a potential therapeutic approach to prevent AKI-to-CKD transition.

Similar to previous reports, we observe a population of dedifferentiated and senescence-like cells in chronically injured kidneys (12–14). This phenomenon has been described as dedifferentiation (2), maladaptive repair (3), failed repair (12), epithelial plasticity (44), and partial epithelial-mesenchymal transition (35, 45). The central hypothesis linking these processes together is that the normal repair process is corrupted or insufficient to make a complete recovery, resulting in a buildup of dedifferentiated cells that contribute to the chronic disease. Based on this idea, the same pathways that trigger dedifferentiation and cell division following AKI also promote CKD, and dedifferentiation is a result of a failure to redifferentiate (2, 6, 8, 9, 35, 45). Our results suggest, however, that dedifferentiation in CKD may represent a fundamentally different pathway than dedifferentiation following AKI. We identify CG1 and CDK5 as factors that are not necessary for homeostasis or repair following AKI but play major roles in dedifferentiation of PTCs and fibrosis in models of CKD. These data indicate that dedifferentiation observed in CKD is not simply a disruption or failure of the adaptive repair process, but potentially an active process governed by distinct cellular programs (Figure 7). These data are in line with recent single-cell RNAseq/snRNAseq studies demonstrating a distinct population of PTCs that arise in CKD (12, 13) and suggest CG1 and CDK5 play a role in the formation of these cells. This is significant because it suggests that therapeutics can be developed to target dedifferentiation specifically in the chronic phase of injury, potentially through targeting CG1/CDK5, to specifically treat CKD.

Another commonly observed phenomenon in CKD is the activation of senescence-like characteristics in PTCs, particularly in G_2_/M-arrested PTCs (8, 46, 47). These PTCs upregulate markers of senescence (including p16, p21, and β-galactosidase), exhibit an increased secretory phenotype, and increased cell size. However, these cells also upregulate cell cycle markers, such as Ki-67, indicating they are not fully senescent (46, 48–50). Our results also highlight a potential tendency for PTCs to acquire senescence-like traits in the presence of CG1, including increased cell size, secretion of profibrotic factors, and β-galactosidase activity. Unlike WT cells, cells lacking CG1 and treated with PAC fail to develop these senescence-like characteristics. While it is unclear to what degree these senescence-like characteristics contribute to CKD, the present data indicate they are a result of dedifferentiation instead of G_2_/M arrest and may represent a defining characteristic of maladaptive dedifferentiation versus adaptive dedifferentiation.

In this study, we utilized 2 different genetic animal models to characterize the role of CG1 and CDK5 in CKD, CG1 global KO and *CDK5^fl/fl^* *Six2*-*Cre*, as well as confirmatory experiments in immortalized cell lines and primary PTCs. While the data from these models consistently demonstrated that CG1 and CDK5 contributed to kidney fibrosis and CKD, these models are not without limitations. One limitation is the use of *Six2* as a driver for Cre recombinase in *CDK5^fl/fl^* mice. *Six2* is expressed as early as 10.5 days post coitum, and the *Six2*^+^ metanephric mesenchyme gives rise to podocyte, proximal, and distal tubule structures up to the connection with the collecting duct (39). Thus, while data from this model suggest CDK5 is involved in the development and progression of renal fibrosis, it does not rule out potential consequences of developmental CDK5 deletion or identify the specific nephron segment where CDK5 induces a profibrotic response. Future studies are necessary to clarify the role of CDK5 in AKI-to-CKD transition. Another limitation is that CG1 is not expressed solely by PTCs. While the majority of CG1 is expressed by KIM-1^+^ PTCs, it is also expressed in other tubule segments and some nontubular cells. Given that the current study utilized a global KO model, it remains possible that CG1 expression in the other tubule segments or nontubule cells contributes to fibrosis and CKD. Future studies are needed to determine the role of CG1 in each of these cell populations. In this study, we also made use of an in vitro model of CKD using primary PTCs treated with AA for 7 days. While no current in vitro model completely recapitulates the complexity of mammalian CKD, our group and others ha6ve demonstrated that this model can recapitulate some of the features of CKD observed in PTCs, namely dedifferentiation, G_2_/M arrest, and secretion of profibrotic cytokines (15, 51, 52). Therefore, we find this model useful for confirming the specific effects of genes or drugs on PTCs. While these individual models are not without limitation, taken together, the data from these models suggest a role of CG1 and CDK5 in CKD.

Here we demonstrate G_2_/M arrest can be uncoupled from dedifferentiation and fibrosis progression through the deletion of *CDK5*, which is regulated by CG1. While CG1-KO or *CDK5*^ΔTub^ mice have the ability to undergo PTC dedifferentiation and repair following AKI, they have reduced maladaptive dedifferentiation during the chronic phase of injury, which plays a role in the progression of fibrosis and CKD. Pharmacological induction of G_2_/M arrest in the absence of dedifferentiation is not sufficient to overcome this protective phenotype. While additional studies are needed to delineate the interplay between dedifferentiation and cell cycle arrest pathways, this study demonstrates the therapeutic potential of targeting these pathways in the treatment of CKD.

## Methods

### Animals.

Animals were fed ad libitum and housed at constant ambient temperature in a 12-hour light cycle. CG1-KO mice were provided by the NIH on a C57BL/6J;129S1/SyImJ background. Heterozygous mice were bred to generate WT and CG1-KO mice. CG1-KO mice were previously reported to have no kidney or developmental phenotypes (27). Conditional *CDK5^fl/fl^* mice were obtained from The Jackson Laboratory and bred with mice expressing Cre recombinase under the *Six2* promotor, also from The Jackson Laboratory. Littermate controls were used for animal studies. Eight- to 12-week-old male mice (20 to 25 g) were used for this study. AA nephropathy was induced in WT and CG1-KO mice by intraperitoneal injection of 3 doses of AA (5 mg/kg body weight; Sigma-Aldrich, A5512) dissolved in PBS. The normal control mice were injected with the same amount of PBS. Blood was collected on the day before the third injection of AA, day 3, day 7, and once a week after that until day 42. Mice were sacrificed and kidneys were isolated on days 3, 7, 14, 21, 28, and 42. For the Rep low cis cisplatin model, cisplatin was dissolved in sterile saline (KD Medical, RGC-3290). WT and CG1-KO mice were injected intraperitoneally with cisplatin at 5 mg/kg once a week for 4 weeks. The same amount of saline was administrated as control. The mice were sacrificed, and kidneys were removed on day 49 following the first dose of cisplatin. Body weight was measured and blood was collected every week from the beginning of the experiments to day 49. For UUO, mice were anesthetized with isoflurane using a Low-Flow Anesthesia System (Kent Scientific, 13-005-111) and body temperature was continuously maintained at 37°C during the surgery. The left kidney was exposed by dorsal incision and the ureter was ligated with silk sutures at 2 points close to the renal pelvis. The mice were sacrificed and both obstructed and contralateral kidneys were removed on day 9. For cisplatin-induced AKI, mice were administrated cisplatin or sterile saline (sham control mice) at 20 mg/kg body weight in a single intraperitoneally injection. Mice were sacrificed on day 5 after injection of cisplatin, and kidneys and blood were collected.

### Antibodies.

The following antibodies were used for immunofluorescent staining and Western blot analysis.

KIM-1 (R&D Systems, AF1817; RRID:AB_2116446), Na^+^/K^+^-ATPase (DSHB, a5; RRID:AB_2166869), SOX9 (Cell Signaling Technology, 82630; RRID:AB_2665492), vimentin (GeneTex, GTX100619; RRID:AB_1952557), α-SMA (Cell Signaling Technology, 19245; RRID:AB_2734735), E-cadherin (Thermo Fisher Scientific, 13-1900; RRID:AB_2533005), cleaved caspase 3 (Cell Signaling Technology, 9664; RRID:AB_2070042), p-CDK5 (Tyr15) (Sigma-Aldrich, SAB4504276), total CDK5 (Thermo Fisher Scientific, AHZ0492; RRID:AB_2536380), ZO-1 (Sigma-Aldrich, AB2272), and collagen type 4 (Millipore, CP56-500UL; RRID:AB_212832) were used as primary antibodies. For immunofluorescent staining, Cy3–donkey anti–mouse IgG antibody (Jackson ImmunoResearch Labs, 715-166-151; RRID:AB_2340817), DyLight 488–donkey anti–mouse IgG antibody (Jackson ImmunoResearch Labs, 715-486-151; RRID:AB_2572300), Cy3–donkey anti–mouse IgG antibody (Jackson ImmunoResearch Labs, 711-166-152; RRID:AB_2313568), DyLight 488–donkey anti–rabbit IgG antibody (Jackson ImmunoResearch Labs, 711-485-152; RRID:AB_2492289), and Alexa Fluor 647–donkey anti–goat IgG antibody (Jackson ImmunoResearch Labs, 705-606-147; RRID:AB_2340438) were used as secondary antibodies. Anti–rabbit IgG antibody conjugated with HRP (Cell Signaling Technology, 5127; RRID:AB_10892860) and anti–mouse IgG antibody conjugated with HRP (Cell Signaling Technology, 5127; RRID:AB_10892860) were used for Western blotting. Anti-GFP antibody (Abcam, ab101863; RRID:AB_10710875) was used to detect GFP protein in CDK5-GFP–transfected cells after fixation with 4% paraformaldehyde.

### Renal function.

Plasma BUN was measured with a QuantiChrom Urea Assay Kit (BioAssay Systems) using the manufacturer’s instructions.

### In situ hybridization.

In situ hybridization was performed according to the manufacturer’s protocol (ACDBio, RNAscope Multiplex Fluorescent Assay v2). Briefly, paraffin-embedded kidney sections of 4 to 6 μm thickness were deparaffinized, incubated with H_2_O_2_ to inhibit endogenous peroxidase activity, and RNA was retrieved followed by incubation with proteinase to allow access to target RNA. The sections were hybridized for *Ccng1* and *Hnf4a* for 2 hours at room temperature and then incubated with appropriate fluorescent probes to visualize the target probe RNA. The images were obtained with a Zeiss LSM 710 confocal microscope and RNA dots were counted by ImageJ (NIH).

### Immunofluorescent staining.

Paraffin-embedded kidneys were sectioned at 4 to 6 μm and subjected to antigen retrieval with high temperature (120°C) and high pressure in TE buffer (pH 9.0) using a pressure cooker. Sections were then incubated with TBS-T containing 1% bovine serum albumin, 0.1%–0.5% Triton X-100, and 3%–5% donkey serum. Sections were then incubated with primary antibodies against the indicated protein of interest overnight, followed by appropriate fluorescence-conjugated secondary antibodies (Jackson ImmunoResearch). For immunocytochemistry, cultured cells were fixed in 4% paraformaldehyde for 20 minutes at room temperature, permeabilized in TBS-T containing 1% bovine serum albumin, 0.1% Triton X-100, and 3% donkey serum, and incubated with primary antibodies for 1 hour, followed by appropriate fluorescence-conjugated secondary antibodies (Jackson ImmunoResearch). Stained whole-kidney sections were scanned at ×20 magnification under polarized light using a Nikon Tie2 eclipse microscope and all images were stitched to create large-scan images using NIS Elements software. Positive area was quantified using thresholding in NIS Elements software.

### Fibrosis quantification.

Kidney sections were stained with picrosirius red and analyzed using Nikon NIS Elements software. Briefly, paraffin-embedded kidney sections, 4 to 6 μm thickness, were incubated with picrosirius red (Electron Microscopy Sciences, 26357) for 2 hours at room temperature following the manufacturer’s protocol, and nuclei were stained with Weigert’s iron hematoxylin (Electron Microscopy Sciences, 26044-05 and 26044-15) for 10 minutes at room temperature. Stained whole-kidney sections were scanned at ×20 magnification under polarized light using a Nikon Tie2 eclipse microscope and all images were stitched to create large-scan images using NIS Elements software. Type I and III collagen fibers are visualized as green and orange when observed by polarized-light microscopy (53) and collagen^+^ area was quantified using NIS Elements software. The renal papilla was omitted from quantification, as it contains collagen-rich vascular structures, which skews the results. The percentage of collagen^+^ area/kidney cortex was defined as the collagen^+^ area divided by kidney cortex area.

### Cell culture.

LLC-PK1 cells (ATCC) were maintained in minimum essential medium (MEM) supplemented with 3% FBS. Mouse renal PTCs were developed as previously described (54). Cells were maintained at 33°C in DMEM/F-12 containing 2.5% FBS and IFN-γ and then transferred to 37°C for differentiation before use (54). The HEK293T (ATCC) cells were maintained in DMEM supplemented with 10% FBS. Cells were treated with AA (2.5–5 μg/mL; Sigma-Aldrich, A5512), PAC (10 μM; Sigma-Aldrich, T7402), or respective vehicles in complete medium for 48 hours. Primary PTCs were isolated from WT mice or CG1-KO mice as described previously (55). In brief, kidney cortex was minced, digested with collagenase (Worthington Biochemical Corporation) and trypsin inhibitor (Worthington Biochemical Corporation), and centrifuged in 32% Percoll medium to purify PTCs. Primary PTCs were then plated in collagen-coated dishes and maintained in DMEM/F-12 medium supplemented with insulin, transferrin, selenium, 0.05 μM hydrocortisone, and 50 μM vitamin C. To avoid changes in PTC phenotype due to repeated passages, these cells were plated as soon as they became confluent for experiments. Primary PTCs were treated with AA at a concentration of 5 to 10 μg/mL for the times indicated.

### Transfection.

Transfections were carried out using Lipofectamine 2000 Transfection Reagent (Thermo Fisher Scientific, 11668019) or Lipofectamine Stem Transfection Reagent (Thermo Fisher Scientific, STEM00003) as per the manufacturer’s protocol on cells grown on glass coverslips for immunofluorescence or cell culture grade plates for other experiments. Cells were plated 1 day prior to transfection to obtain 60% to 70% confluence.

### Western blot analysis.

M-PER Mammalian Protein Extraction Reagent (Thermo Fisher Scientific, 78501) along with protease and phosphatase inhibitors were used to lyse the cells. Mouse kidney cortex was lysed in SDS lysis buffer (10 mM Tris-HCl, 2 mM EDTA, 1% SDS) or T-PER Tissue Protein Extraction Reagent (Thermo Fisher Scientific, 78510). Protein (20 μg per sample) was loaded on SDS polyacrylamide gels and transferred onto polyvinylidene difluoride membranes. Membranes were incubated overnight with primary antibodies as described below followed by appropriate HRP-conjugated secondary antibodies. The images were obtained using the ChemiDoc MP Imaging System (Bio-Rad) and the band intensity was analyzed with Image Lab software (Bio-Rad). See complete unedited blots in the supplemental material.

### Immunoprecipitation.

Myc-tagged mouse CG1 plasmid, pCMV-6Myc(+Asc1)-M Cyclin G1 (gift from N. Yabuta and N. Nojima, Osaka University Research Institute for Microbial Diseases, Japan), was transfected into HEK293T cells using Fugene-6 (Promega) as per the manufacturer’s protocol. Forty-eight hours after transfection, cells were lysed with M-PER containing protease inhibitors. Anti-Myc (9E10) antibody (VAPR9E10, Vanderbilt University Core) was used to immunoprecipitate CG1 from 200 μg of whole-cell lysate overnight at 4°C. Fifty microliters of a 50% slurry of Protein A/G agarose beads (Thermo Fisher Scientific) was used to capture the precipitate, washed 3 times with PBS, followed by PBS containing 500 mM NaCl. Beads were then resuspended in SDS sample buffer and further analyzed by Western blotting for Myc-CG1 and CDK5.

### Real-time PCR analysis.

Total RNA was extracted from the kidney cortex using an RNeasy Mini kit (QIAGEN, 74106) according to the manufacturer’s protocol and was used to synthesize cDNA using an iScript cDNA synthesis kit (Bio-Rad, 1708891). Quantitative real-time PCR (RT-PCR) was performed with iTaq Universal SYBR Green (Bio-Rad, 1725121). The relative mRNA expression of each gene was calculated using the ΔΔCt method. Primers used are listed in Supplemental Table 2.

### Human kidney organoids.

Human kidney organoids were derived from WA09 (H9) or Ai9 human pluripotent stem cells or via directed differentiation in thin-layer adherent cultures as previously described (28). Organoids were treated with 50 μM cisplatin in culture media for 48 hours or uninjured. Organoids were then whole-mount fixed by adding 8% paraformaldehyde (Electron Microscopy Sciences) in PBS to an equal amount of culture media for 15 minutes at room temperature. Organoids were kept in the plate for staining and imaging. After fixation, samples were blocked in 5% donkey serum with 0.3% Triton X-100 in PBS, incubated with primary antibodies and biotinylated LTL in PBS with 3% donkey serum overnight, and incubated with appropriate Alexa Fluor–conjugated secondary antibodies or Alexa Fluor 647–streptavidin with DAPI. Confocal optical sections were acquired on a Nikon A1R scanning laser confocal microscope (ISCRM Garvey Core, University of Washington). LTL immunofluorescence was pseudocolored red for merged images.

### Human samples.

Deidentified human samples were obtained from 9 patients undergoing partial or radical nephrectomy for urological indication. KIM-1 staining was used to confirm PTC injury and the samples were divided into 2 groups based on the presence or absence of KIM-1. Interstitial fibrosis was evaluated by a trained senior pathologist and tissues with less than 10% interstitial fibrosis were categorized as normal kidney and the others were classified as fibrotic kidney (Supplemental Table 1).

### Statistics.

All statistical analyses were performed using GraphPad Prism 7 software. All data are presented as mean ± SD. Unpaired, 2-tailed *t* test or 1-way ANOVA followed by Tukey’s post hoc test was used to assess the differences. A *P* value of less than 0.05 was considered statistically significant.

### Study approval.

All animal experiments were approved by the Institutional Animal Care and Use Committee of Vanderbilt University Medical Center. Human samples were obtained under protocols approved by the Institutional Review Board at Boston University Medical Center and Brigham and Women’s Hospital. All necessary patient/participant written informed consent was obtained.

## Author contributions

KT and BCE contributed equally to the work and should be considered co–first authors. First author order was decided based on reverse alphabetical order. KT, BCE, S Sugahara, S Sant, and CRB designed the experiments, performed experiments, collected data, and analyzed the data. BSF performed organoid culture experiments and aided manuscript preparation. SSW and SMP provided biopsy samples and helped with tissue staining and manuscript preparation. AP, RZ, and RCH helped with experimental design and manuscript preparation.

## Supplementary Material

Supplemental data

## Version 1 12/01/2022

Electronic publication
