# Supplementary material for: Cyclin G1 induces maladaptive proximal tubule cell dedifferentiation and renal fibrosis through CDK5 activation
Source: J Clin Invest. 2022 Dec 1;132(23):e158096. doi: 10.1172/JCI158096 (PMC9711881; doi:10.1172/JCI158096)

Supplemental Material

Cyclin G1 induces maladaptive proximal tubule cell dedifferentiation and renal fibrosis through CDK5 activation

Kensei Taguchi et al.

Supplemental Material Table of Contents:

Legends of Supplemental Figures

Supplemental Figure 1

Supplemental Figure 2

Supplemental Figure 3

Supplemental Figure 4

Supplemental Figure 5

Supplemental Figure 6

Supplemental Figure 7

Supplemental Table 1

Supplemental Table 2

Supplemental Figure 1

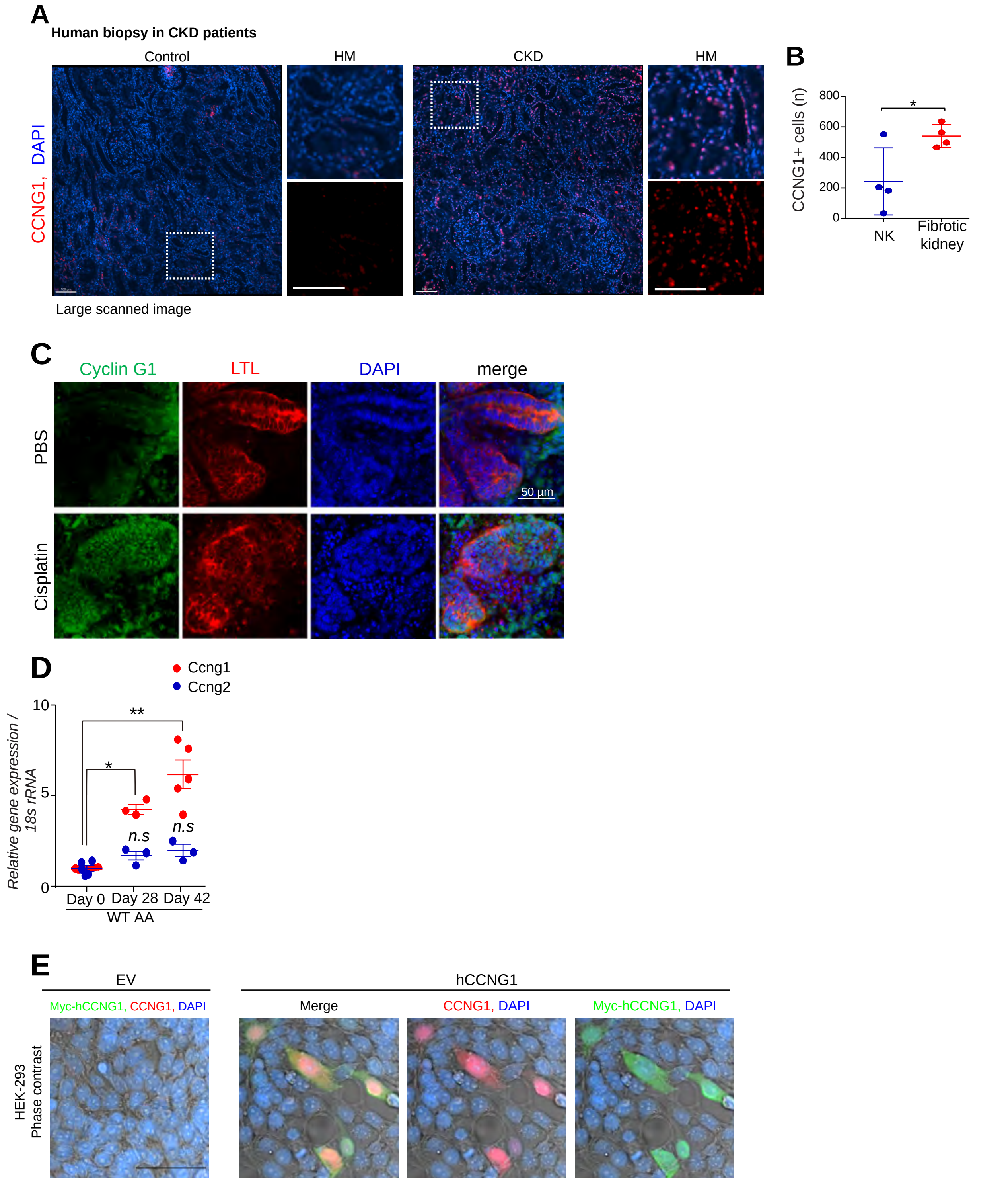

Supplemental figure 1. CG1 is upregulated in response to injury in human biopsies and human kidney organoids. (A) Representative large scan images of patient biopsies labeled with CG1 (red) as in Figure 1G. Scale bar: 100 μm. (B) Quantification of CCNG1+ area / one field. n = 5. (C) Representative confocal images of kidney organoids derived from inducible pluripotent stem cells, treated with cisplatin, and stained for CG1 and LTL. Scale bar: 50μm. (D) Real Time PCR analysis of *Ccng1* (CG1) and *Ccng2* (cyclin G2) expression at 0, 28, and 42 days after AA injection. (E) Representative images of Myc-CG1 transfected HEK-293 cells stained with antibodies against CG1 and Myc. Scale bar: 100 μm. Data are presented as the mean ± SD. Unpaired t-test was performed (B) and one-way ANOVA and subsequent Tukey's post-hoc test was carried out to identify statistical difference (D).*P<0.05, **P<0.01.

Supplemental Figure 2

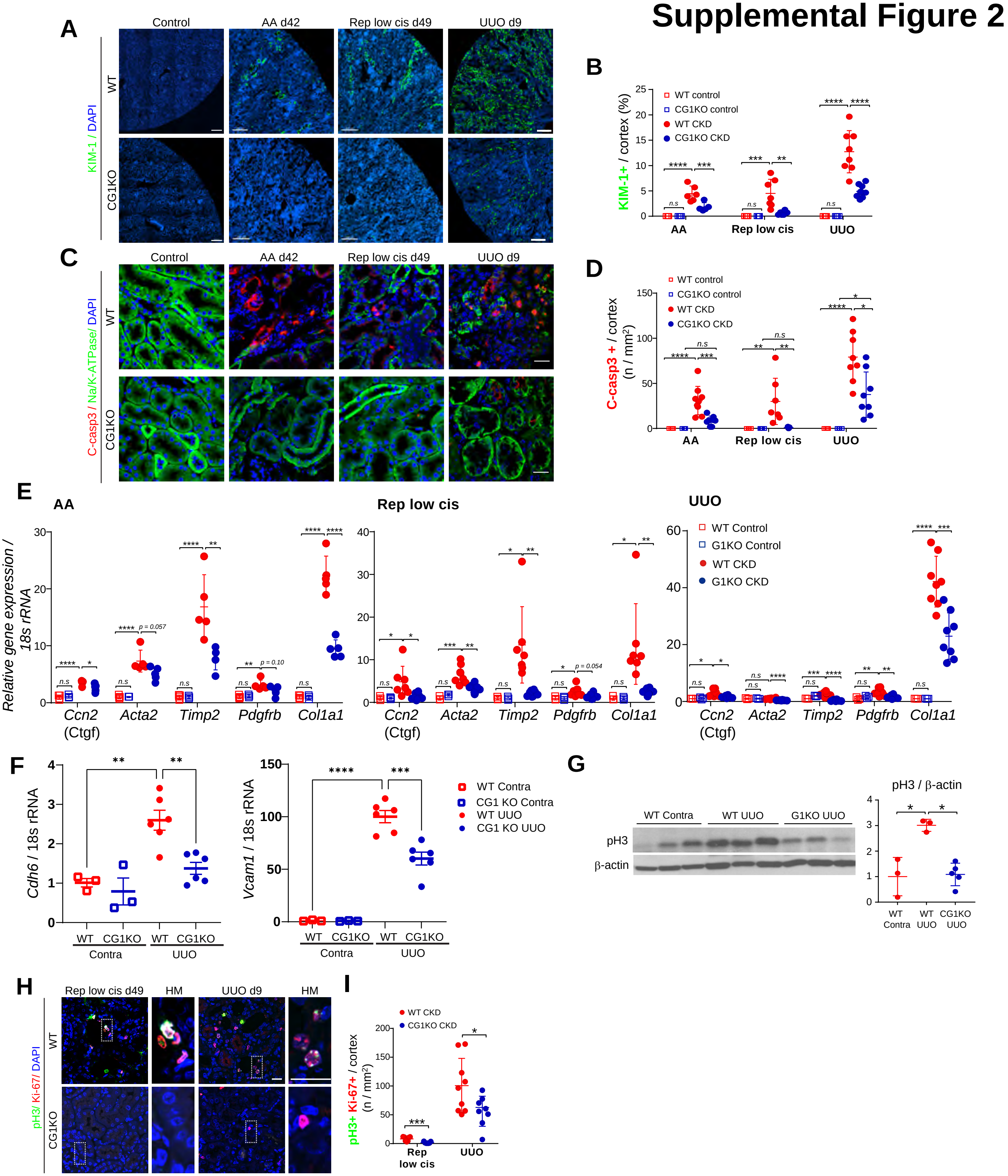

Supplemental figure 2. PTCs CG1 protects agains tubular damage, apoptosis, and G2-M phase arrest in CKD. (A) Representative large, scanned images of KIM-1-labeled kidneys of WT and CG1KO mice in following AA, rep low cis, or UUO. Scale bar: 200 μm. (B) The corresponding quantitation of KIM-1+ area / cortex (%) following AA rep low cis, or UUO. Control (n = 5) and injured kidney (n = 8-10). (C) Representative images of C-casp3 and Na/K-ATPase-stained kidney sections from WT and CG1KO mice in control kidney following AA, rep low cis, or UUO. Scale bar: 20 μm. (D) Quantitative analysis for number of C-casp3+ PTCs / cortex. Control (n = 5) and injured kidney (n = 8-10). (E) Real-time PCR analysis of several profibrotic cytokines and markers in WT and CG1KO mice following AA, rep low cis, or UUO. Control (n = 5) and injured kidney (n = 8). (F) Real-time PCR analysis of Cdh6 and Vcam1 in wild-type and CG1KO mouse kidneys. Contralateral; n = 3, UUO; n = 6. (G) Western blotting for pH3 in whole kidney lysates of WT and CG1KO mice following UUO and quantification of pH3 / β-actin. (H) Representative images of pH3+ Ki-67+ labeled kidneys of WT and CG1KO mice in Rep low cis and UUO. Scale bar: 20 μm. (I) Qualification of the number of pH3+ Ki-67+ PTCs in cortex (n /mm2). Data are presented as the mean ± SD. One-way ANOVA and subsequent Tukey's post-hoc test were performed to identify statistical difference. *P<0.05, **P<0.01, ***P<0.001, and ****P<0.0001.

Supplemental Figure 3

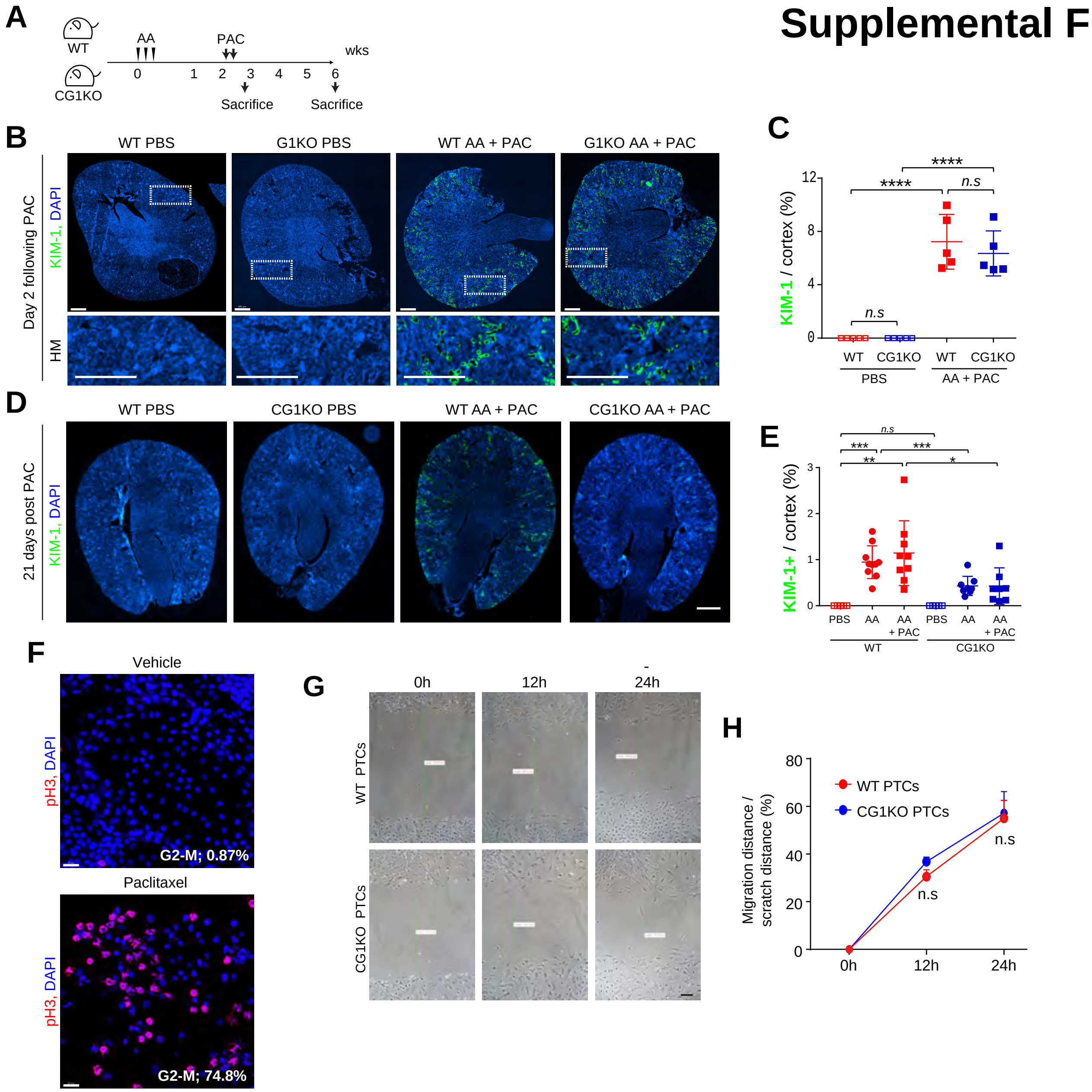

Supplemental figure 3. CG1KO mice developed acute tubular injury to the same degree as WT mice following paclitaxel administration (A) A schematic diagram of combination treatment of AA and PAC in WT and CG1KO mice. (B) Representative large, scanned images of KIM-1-labeled kidneys of WT and CG1KO 2 days after PAC administration. Scale bar; 500 μm. (C) The corresponding quantitation for KIM-1+ area / cortex (%) in Supple 2B. WT PBS, n = 5; CG1KO PBS, n = 5; WT AA + PAC, n = 5; CG1KO AA + PAC, n = 5. (D) Representative large, scanned images of KIM-1-labeled kidneys of WT and CG1KO 3 weeks following PAC injection. Scale bar; 500 μm. (E) The corresponding quantitation for KIM-1+ area / cortex (%) from 3D. WT PBS, n = 5; CG1KO PBS, n = 5; WT AA + PAC, n = 9; CG1KO AA + PAC, n = 8. (F) pH3 staining in LLC-PK1 cells treated with PAC alone. Scale bar: 30 μm. (G) Representative images of scratch wound healing assays in primary WT or CG1KO PTCs. n = 3 independent experiments. Scale bar: 100 μm. (H) Quantification of the scratch assays described in A. Scale bar: 100 μm. One-way ANOVA and subsequent Tukey's post-hoc test was performed to identify statistical difference. *P<0.05, **P<0.01, ***P<0.001, and ****P<0.0001.

Supplemental figure 4

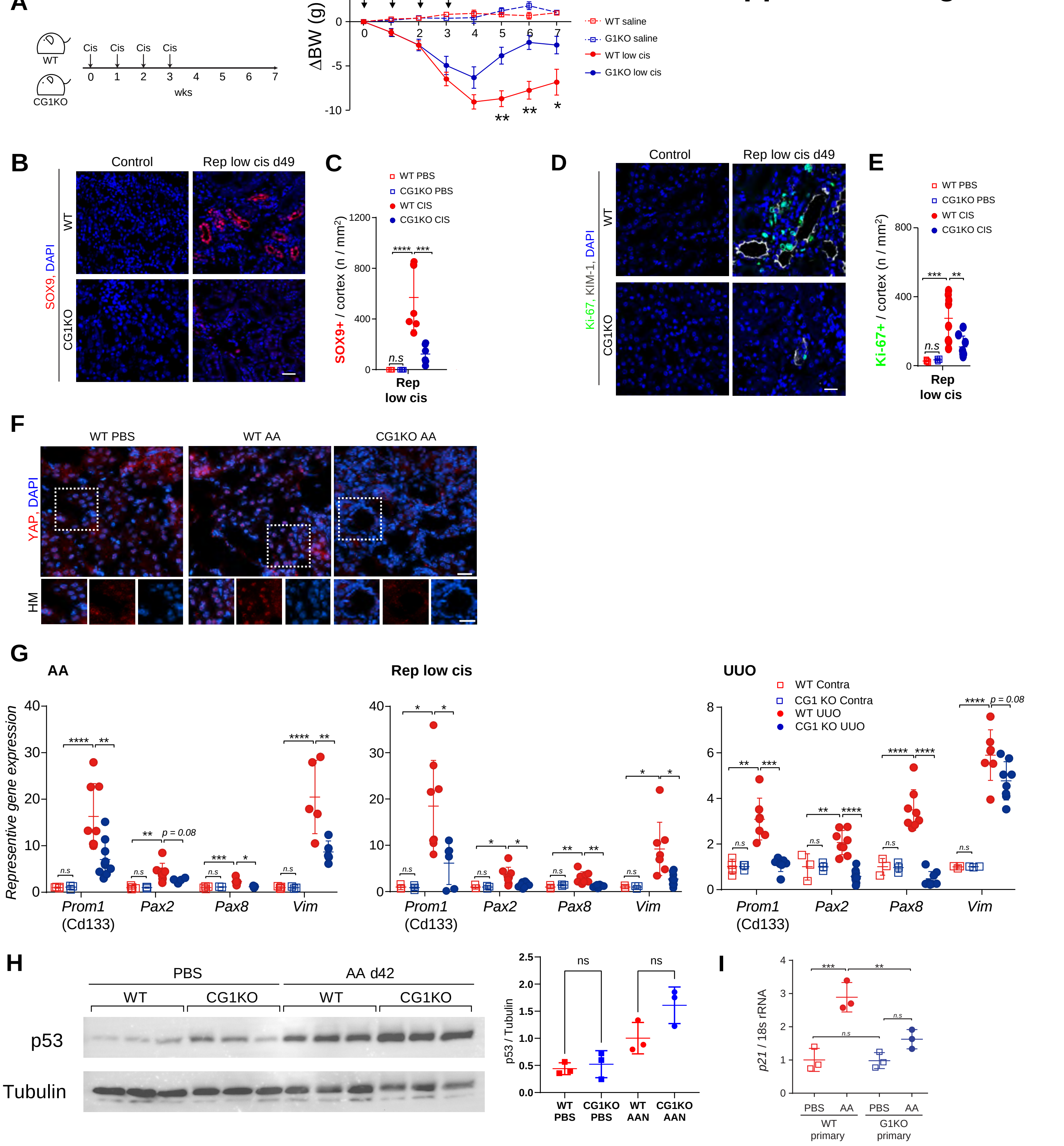

Supplemental Figure 4. CG1KO mice have reduced dedifferentiation marker expression in models of CKD. (A) Change in BW (compared to day 0) at weekly time points in rep low cis model. Data are presented as the mean ± SD. (B) Representative images of SOX9-labeled kidney sections from wild-type and CG1KO mice in rep low cis and UUO. Scale bar: 20 μm. (C) Quantification of the number of SOX9+ cells / kidney. Control; n = 5, injured kidney; n = 8. (D) Representative images of PH3+ Ki67+ PTCs labeled kidneys of WT and CG1KO mice in rep low cis and UUO. Scale bar; 20 μm. (E) Quantification of the number of PH3+ Ki67+ PTCs in cortex (n / mm2). Control (n = 5), CKD (n = 8-9). Scale bar: 20 μm. (F) Representative immunofluorescent images of YAP stained kidneys from wild-type (WT) or CG1KO mice treated with PBS or AA. Scale bar = 20 μm. (G) Real-time PCR for dedifferentiation markers in the chronic phase of AAN (day 42), Rep low cis (day 49), and UUO (day 9). (H) Western blot analysis and quantification of p53 levels in wild-type and CG1KO kidneys 42 days following PBS or AA injection. (I) Real-time PCR analysis of p21 levels in wild-type or CG1KO primary PTCs following AA or PBS treatment. Data are presented as the mean ± SD. One-way ANOVA and subsequent Tukey's post-hoc test were performed to identify statistical difference. *P<0.05, **P<0.01, ***P<0.001, and ****P<0.0001.

Supplemental figure 5

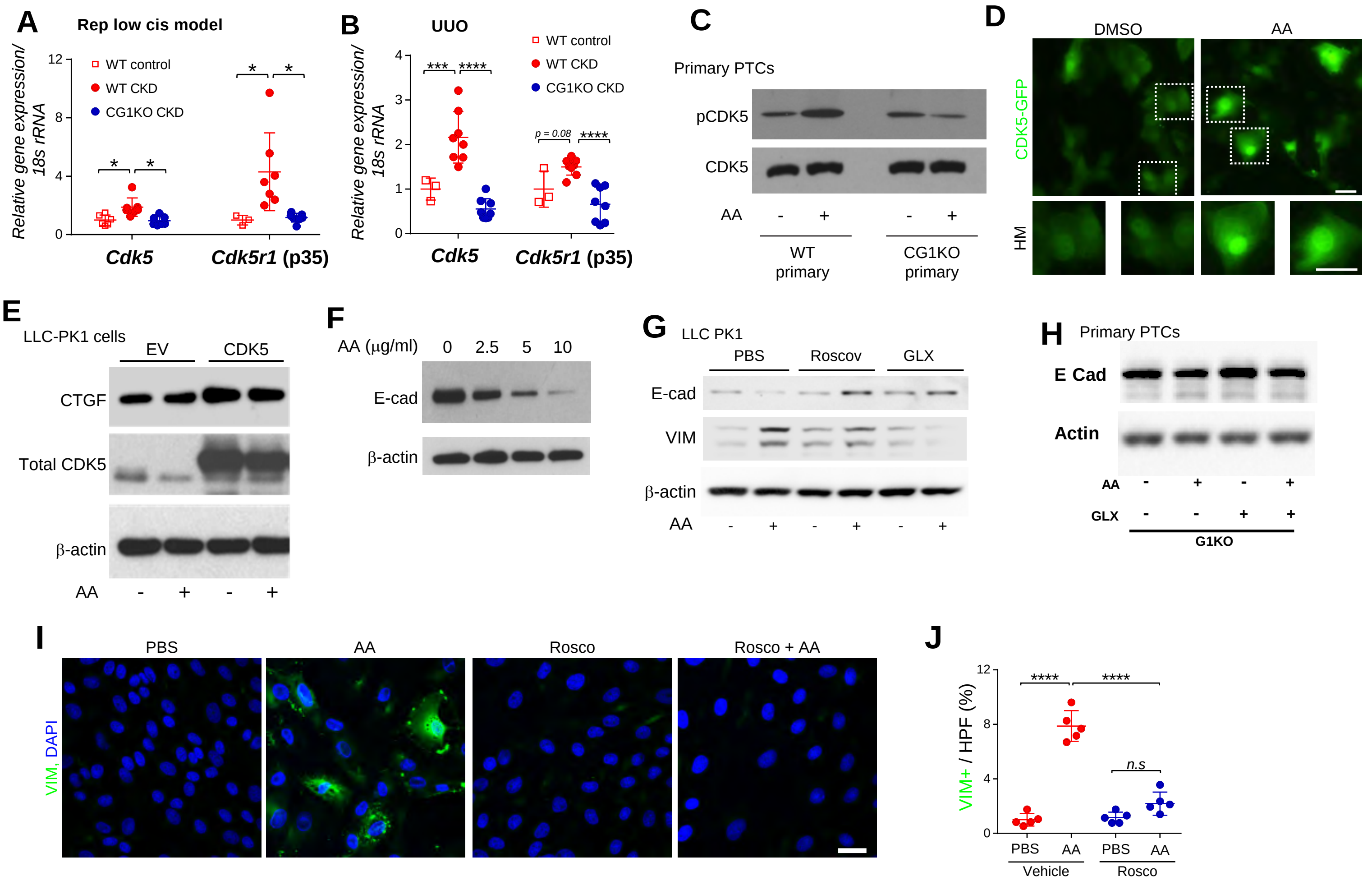

Supplemental figure 5. Inhibition of CDK5 suppresses PTC dedifferentiation. (A and B) Real-time PCR for CDK5 and p35 following rep low cis or UUO models. Control; n = 3-5, injured kidney; n = 5-10. (C) Western blot analysis of pCDK5 and CDK5 in WT or CG1KO primary PTCs with or without AA (2.5 μg/mL). (D) Representative images in CDK5-GFP transfected RPTECs with or without AA (5 μg/ml) for 48hrs. Scale bar: 25 μm. (E) Western blot analysis of CTGF and total-CDK5 in CDK5-overexpressed LLC-PK1 cells with or without AA (2.5 μg/mL). (F) Western blot analysis of E-cadherin in RPTEC treated with increasing doses of AA for 48hr. (G) Western blot analysis of E-cad and VIM from the lysates in Figure 5J (reprobe of same blot with loading control). (H) Western blot analysis of E cad in CG1KO primary PTCs treated with PBS or AA +/- GLX. (I) Representative images of VIM staining in LLC-PK1 cells treated with AA (2.5 μg/mL) with or without Rosco. Scale bar: 20 μm. (J) Quantification of VIM+ area / HPF in Supple Fig 5E. n = 5, respectively. Data are presented as the mean ± SD. One-way ANOVA and subsequent Tukey's post-hoc test were performed to identify statistical difference. *P<0.05, **P<0.01, ***P<0.001, and ****P<0.0001.

Supplemental figure 6

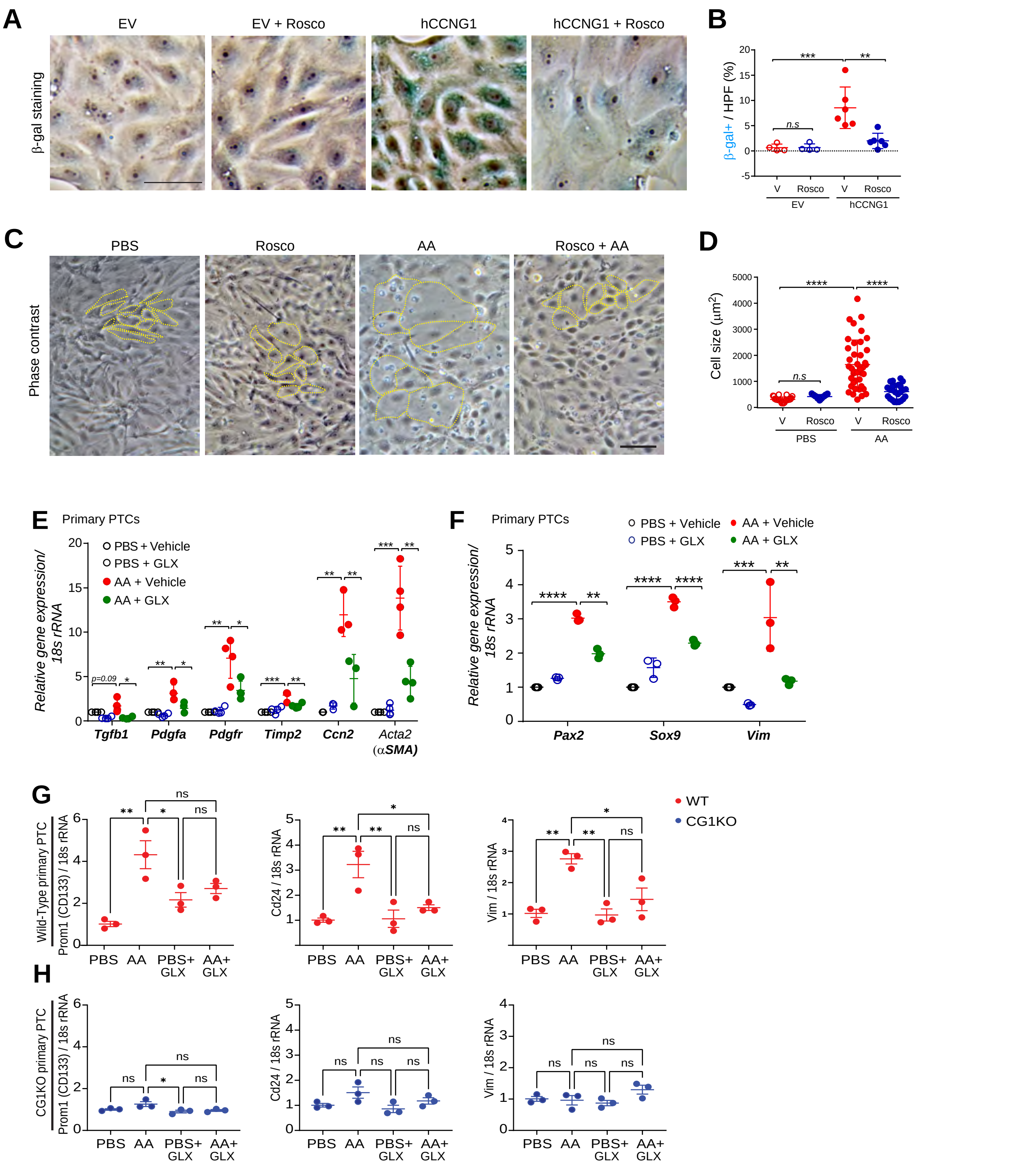

Supplemental figure 6. CDK5 inhibition prevents hCG1 or AA induced cell morphological changes. (A) Representative images of β-galactosidase staining in hCG1-overexpressed LLC-PK1 in the presence or absence of Rosco. For β-galactosidase quantification, areas with similar size cells were chosen so similar numbers of cells could be quantified. Scale bar: 50 μm. (B) Quantification of β-gal+ area / HPF in Supple Fig 5A. n = 4-6, respectively. (C) Representative images of phase contrast in AA-treated LLC-PK1 in the presence or absence of Rosco. A portion of cells are outlined in yellow to demonstrate cell size. Scale bar: 50 μm. (D) Quantification of cell size in experiment described in Supple Fig 5C. n = 25, respectively. (E) Real-time PCR quantification of profibrotic markers in WT primary PTCs treated with AA in the presence or absence of R0. n = 3-4 independent experiments. (F) Real-time PCR for dedifferentiation markers in WT primary PTCs treated with AA (5 μg/mL) for 7 days in the presence or absence of roscovitine or GLX. n = 3 independent experiments. (G and H) Real-time PCR analysis of dedifferentiation markers in WT (G) or CG1KO (H) primary cells treated with PBS, AA, PBS+GLX or AA+GLX. n=3. Data are presented as the mean ± SD. One-way ANOVA and subsequent Tukey's post-hoc test was performed to identify statistical difference. *P<0.05, **P<0.01, ***P<0.001, and ****P<0.0001. V = Vehicle.

Supplemental Figure 7

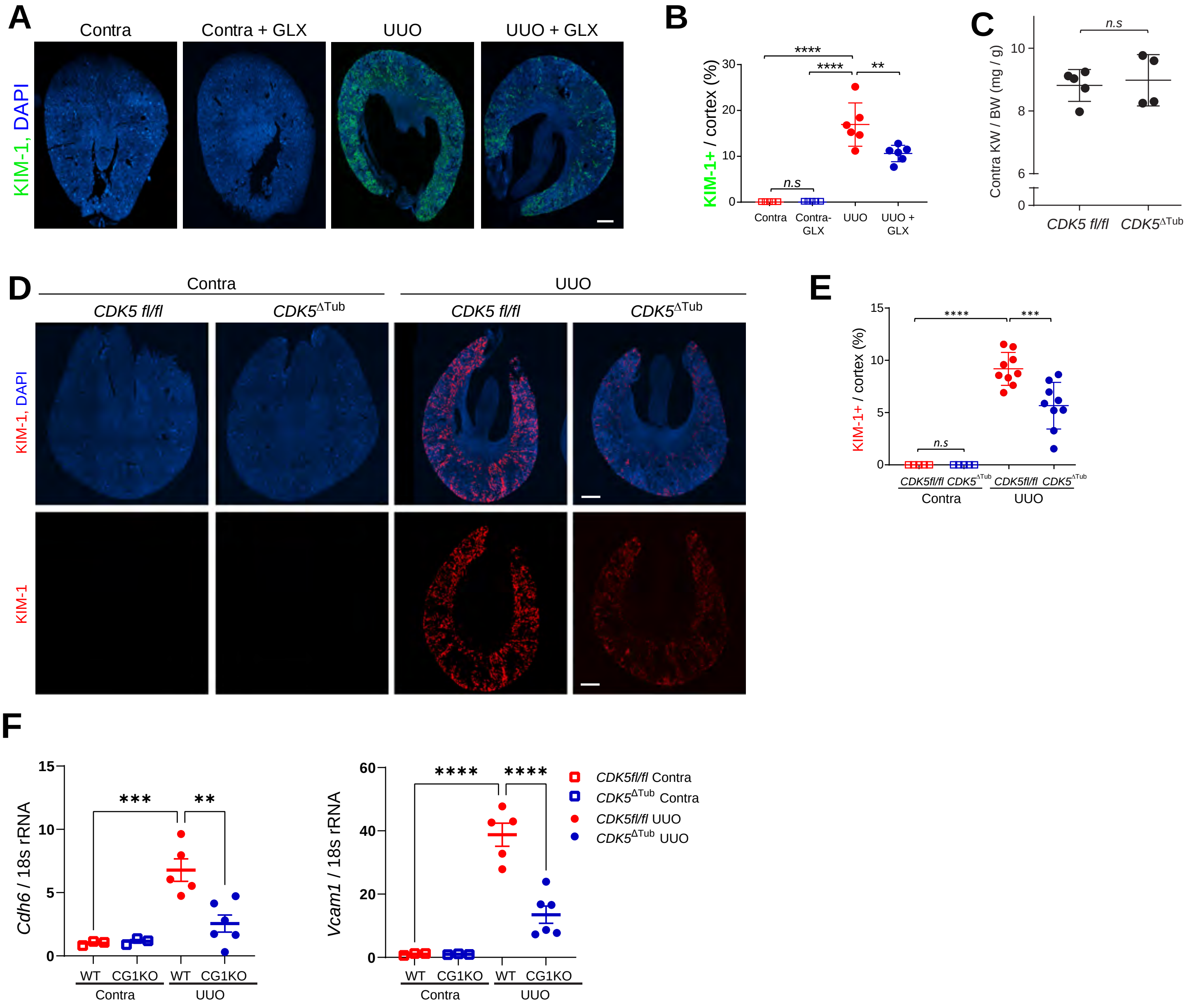

Supplemental Figure 7. Kim-1 is reduced with inhibition or knockout of CDK5 following UUO. (A) Large, scanned images of KIM-1-labeled kidneys from UUO model with GLX or vehicle. Scale bar: 500 μm. (B) The corresponding quantitation of KIM-1+ area / cortex (%). Contralateral kidney of vehicle, n = 5; contralateral kidney of GLX, n = 5; UUO with vehicle, n = 6; UUO with R0, n = 6. (C) Quantification of kidney weight/body weight in contralateral kidneys. n = 4-5 kidneys. (D) Large, scanned images of KIM-1-labeled kidneys from UUO model with CDK5fl/fl or CDK5$^{\Delta Tub}$. Scale bar: 300 μm. (E) The corresponding quantitation of KIM-1+ area / cortex (%). Contralateral kidney of vehicle, n = 5; contralateral kidney of R0, n = 5; UUO with vehicle, n = 9; UUO with R0, n = 9. (F) Real-time PCR analysis of Cdh6 and Vcam1 in CDK5fl/fl and CDK5$^{\Delta Tub}$ mouse kidneys. Contralateral; n = 3, UUO; n = 6. Data are presented as the mean ± SD. Unpaired t-test was performed (C) and one-way ANOVA and subsequent Tukey's post-hoc test was performed (B and E) to identify statistical difference. *P<0.05, **P<0.01, ***P<0.001, and ****P<0.0001.

Supplemental Table1

Sample ID	Ethnicity	Race	Gender	Cr (mg/dL)	Interstitial fibrosis, tubular atrophy	KIM-1
UR70	Unknown / Not Reported	White	Female	1.65	11-25%	+
UR76	NOT Hispanic or Latino	Unknown / Not Reported	Male	3.76	> 50%	+
UR105	NOT Hispanic or Latino	Unknown / Not Reported	Male	0.99	1-10%	-
UR108	NOT Hispanic or Latino	White	Female	0.96	1-10%	-
UR109	Unknown / Not Reported	White	Male	1.04	26-50%	+
UR110	NOT Hispanic or Latino	White	Female	0.77	1-10%	-
UR113	NOT Hispanic or Latino	White	Female	0.92	1-10%	-
UR117	NOT Hispanic or Latino	White	Male	0.86	11-25%	+
UR125	NOT Hispanic or Latino	White	Female	0.66	Unknown / Not Reported	+

Supplemental Table1

Characteristics of patients with or without CKD. CKD, chronic kidney injury; Cr, creatinine; KIM-1, kidney injury molecule-1.

Supplementary Table 2

Primers	Sequence
CCNG1-Forward	5’-CCTTGCCATTTGAGAGGAGA-3’
CCNG1-Reverse	5’-TAGATAGCGCCAGCACAGAA-3’
CCNG2-Forward	5’-GAGATACCAACCTCGGGAAA-3’
CCNG2-Reverse	5’-TCAGTGCCAGATCCAAAGAA-3’
CTGF-Forward	5’-CTTCTGCGATTTCGGCTCC-3’
CTGF-Reverse	5’-TACACCGACCCACCGAAGA-3’
TGF-β-Forward	5’-CAGTGGCTGAACCAAGGAGAC-3’
TGF-β-Reverse	5’-ATCCCGTTGATTTCCACGTG-3’
αSMA-Forward	5’-AGGGCTGGAGAATTGGATCT-3’
αSMA-Reverse	5’-CCAGCAAAGGTCAGAGAAGG-3’
PDGFA-Forward	5’-CAAGACCAGGACGGTCATTT-3’
PDGFA-Reverse	5’-GATGGTCTGGGTTCAGGTTG-3’
PDGFRβ-Forward	5’-CTGTCCGTGTTATGGCTCCT -3’
PDGFRβ -Reverse	5’-GGGACATCTGTTCCCACATC-3’
TIMP-2-Forward	5’-GTTTTGCAATGCAGATGTAG-3’
TIMP-2-Reverse	5’-ATGTCGAGAAACTCCTGCTT-3’
COL1A1-Forward	5’-GTCCCTGAAGTCAGCTGCATA-3’
COL1A1-Reverse	5’-TGGGACAGTCCAGTTCTTCAT-3’
FN-Forward	5’-ACAGAAATGACCATTGAAGG-3’
FN-Reverse	5’-TGTCTGGAGAAAGGTTGATT-3’
CD133-Forward	5’-GAAAAGTTGCTCTGCGAACC-3’
CD133-Reverse	5’-TCTCAAGCTGAAAAGCAGCA-3’
CD24-Forward	5’-CTTCTGGCACTCCTACC-3’
CD24-Reverse	5’-CACATTGGACTTGTGGTTGC-3’
Pax2-Forward	5’-GCGAGCCGACACCTTCAC-3’
Pax2-Reverse	5’-GACGCTCAAAGACTCGATCCA-3’
Pax8-Forward	5’-CAGAAGGCGTTTGTGACAATGA-3’
Pax8-Reverse	5’-TGCACTTTGGTCCGGATGAT-3’
SOX9-Forward	5’-GTGCAAGCTGGCAAAGTTGA-3’
SOX9-Reverse	5’-TGCTCAGTTCACCGATGTCC-3’
VIM-Forward	5’-CCGTCACCTTCGTGAATACC-3’
VIM-Reverse	5’-CCTGCTCTCTTCTCCTTCCA-3’
Snail-Forward	5’-CACACGCTGCCTTGTGTCT-3’
Snail-Reverse	5’-GGTCAGCAAAAGCACGGTT-3’
Twist-Forward	5’-CTGCCCTCGGACAAGCTGAG-3’
Twist-Reverse	5’-CTAGTGGGACGCGGACATGG-3’
Slug-Forward	5’-GAAGCCCAACTACAGCGAAC-3’
Slug-Reverse	5’-CACTGGGTAGAGGAGAGTGGA-3’
ATP1B1-Forward	5’-TCGGGACCATCCAAGTAA-3’
ATP1B1-Reverse	5’-TGATGTTTAGCACGTAGGC-3’
AQP1-Forward	5’-AGGCTTCAATTACCCACTGGA-3’
AQP1-Reverse	5’-GTGAGCACCGCTGATGTGA-3’
CDH1-Forward	5’-GTCTCCTCATGGCTTTGC-3’
CDH1-Reverse	5’-CTTTAGATGCCGCTTCAC-3’

Supplementary Table 2

Primer sequences for Real-time PCR.

Figure 4D

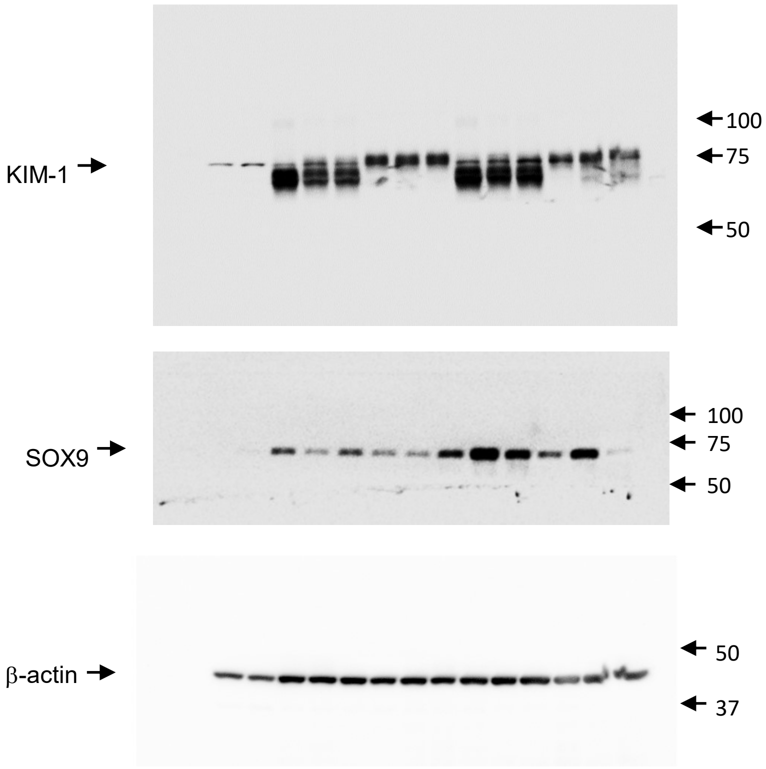

Figure 5B

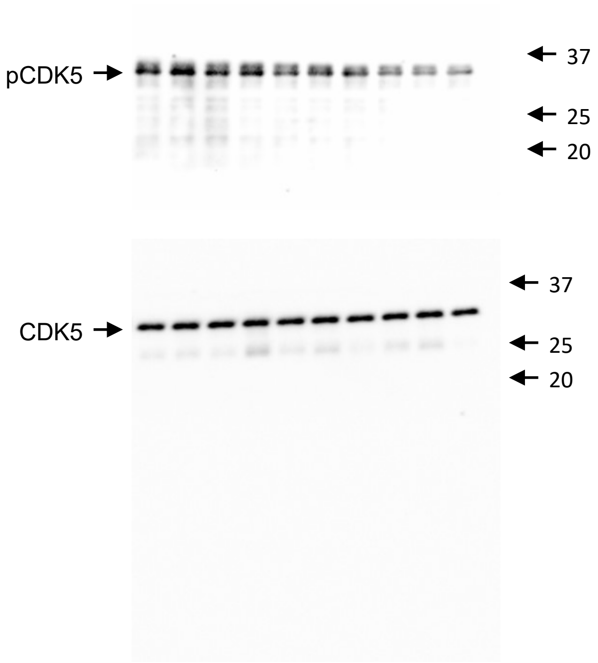

Figure 4H

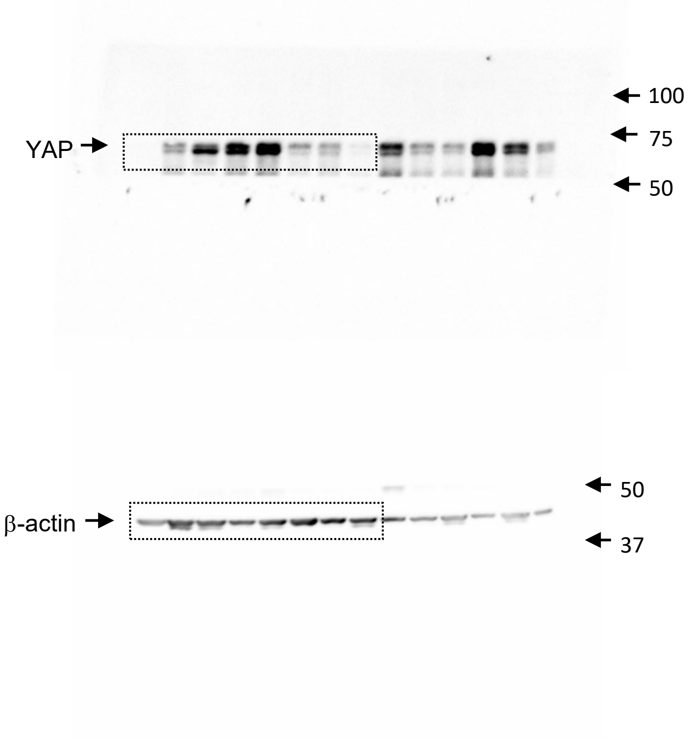

Figure 5G

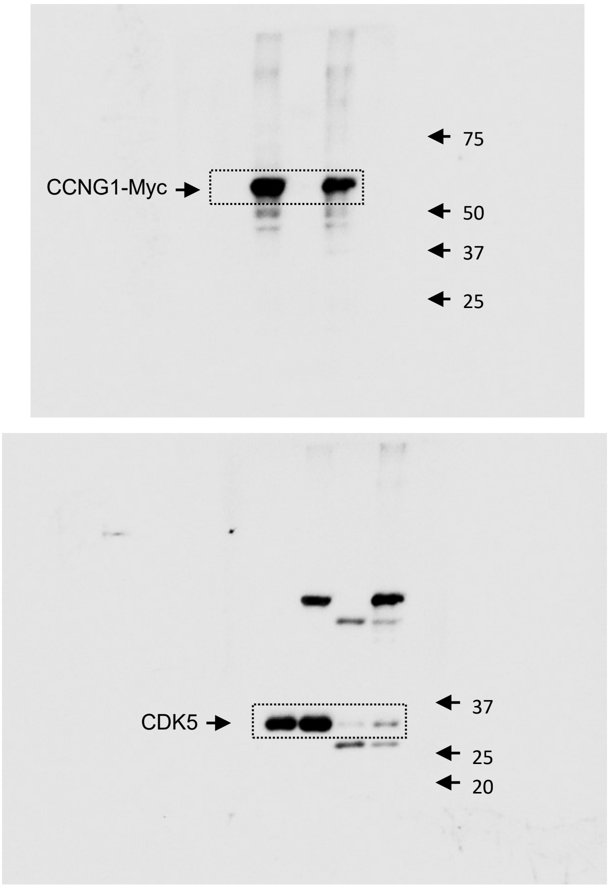

Figure 5H

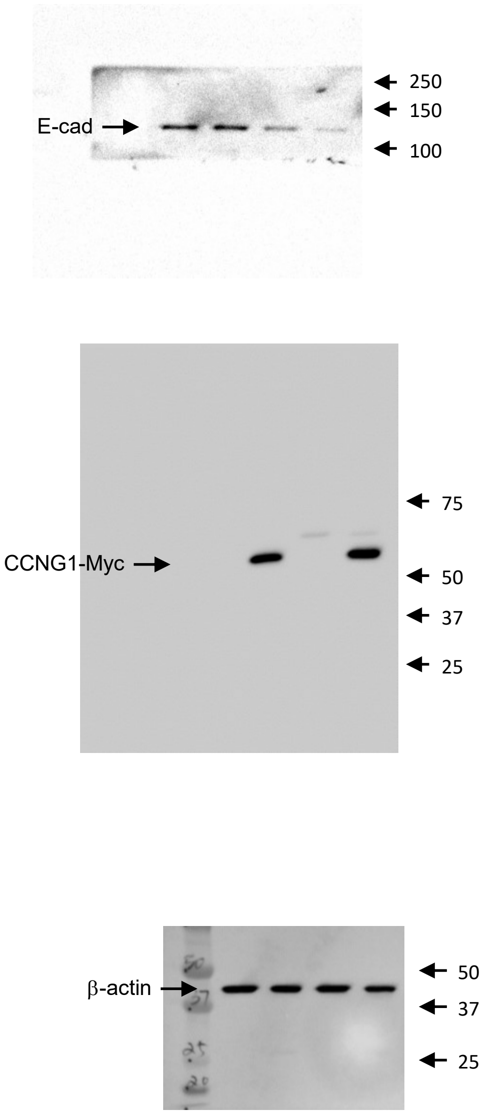

Figure 5J

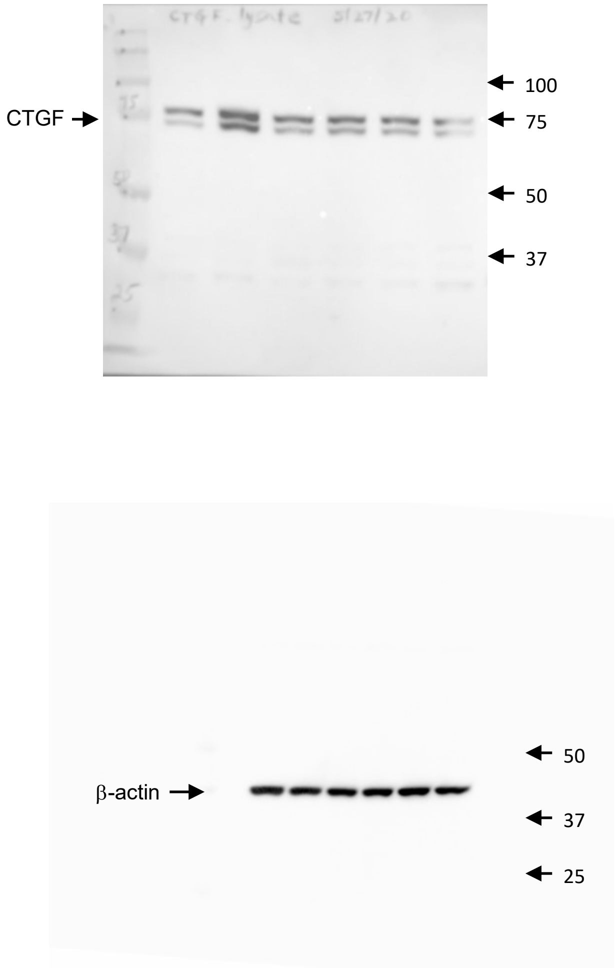

Figure 5K

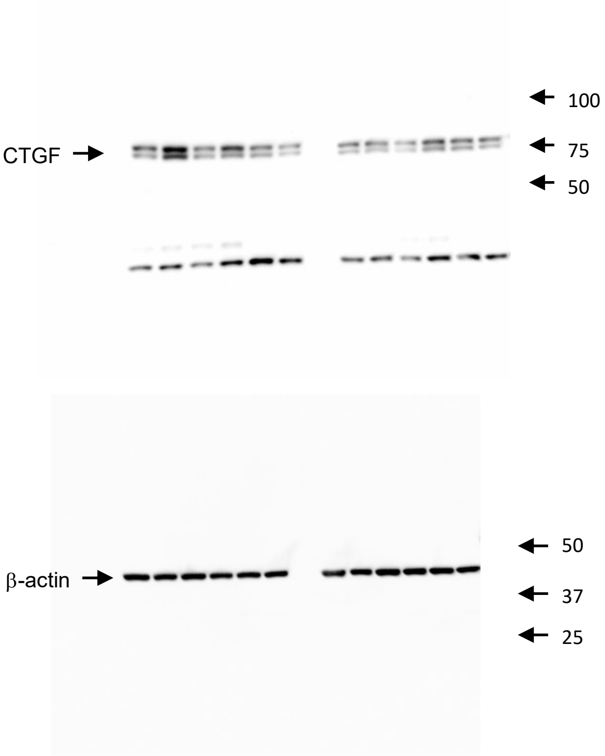

Figure 6E

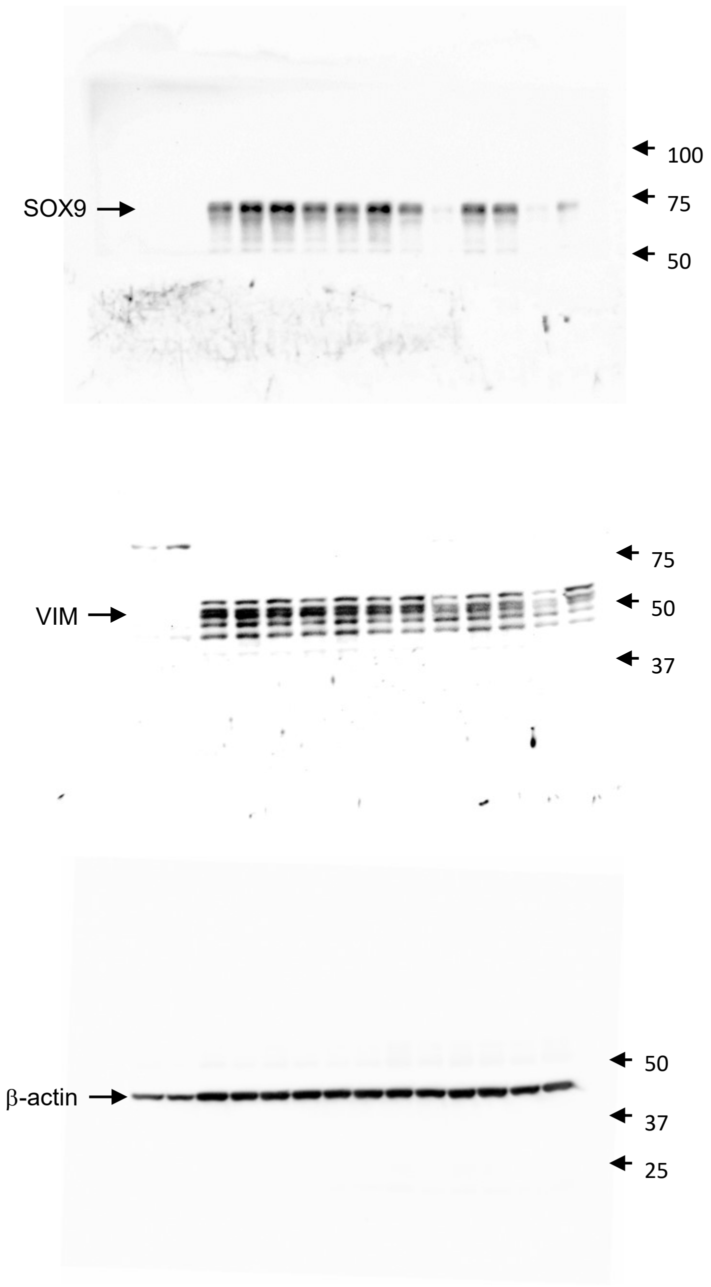

Supplementary figure 2F

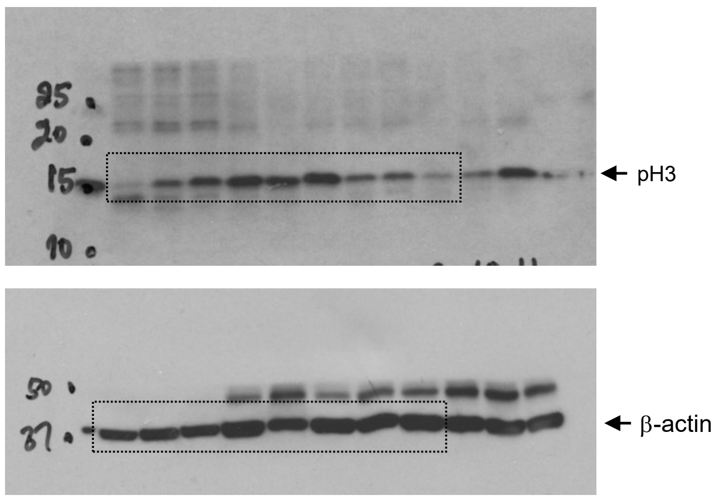

Figure 7A

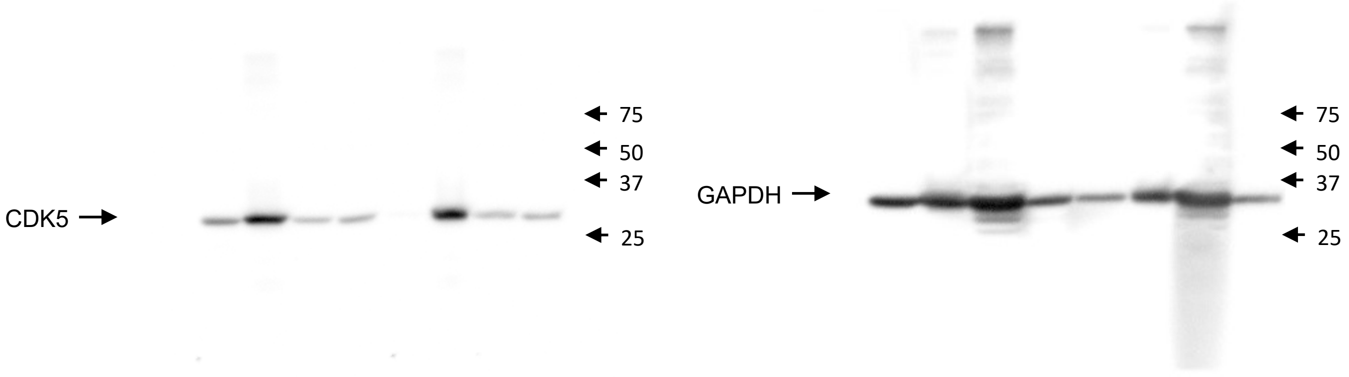

Supplementary figure 4H

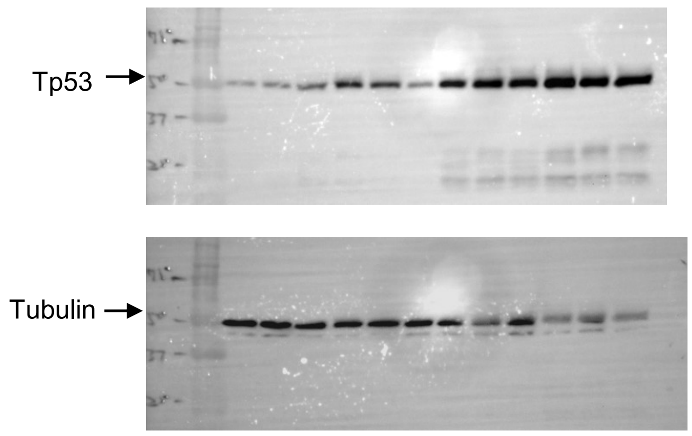

Supplementary figure 5C

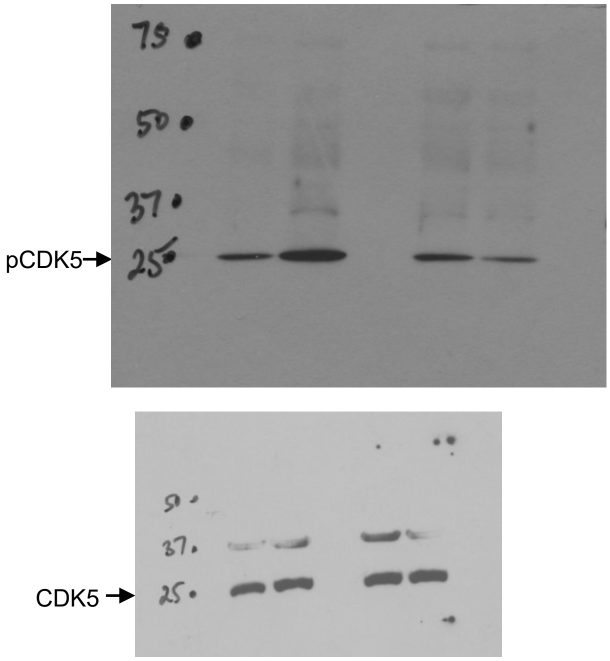

Supplementary figure 5E

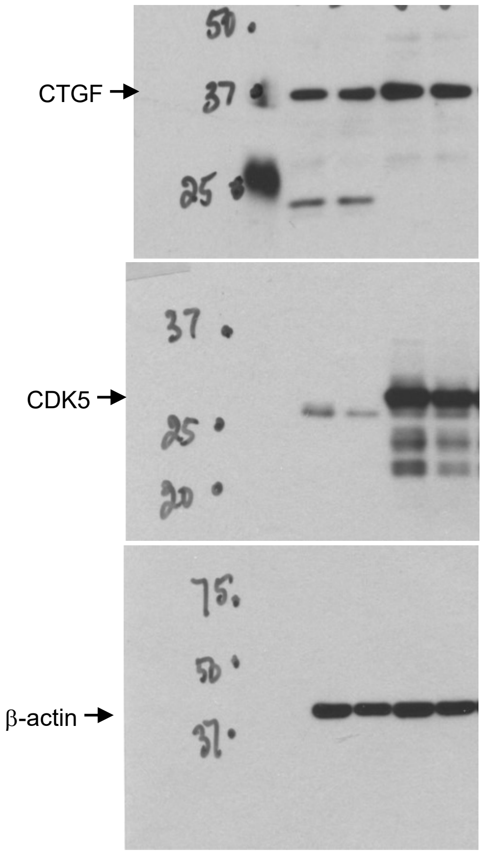

Supplementary figure 5F

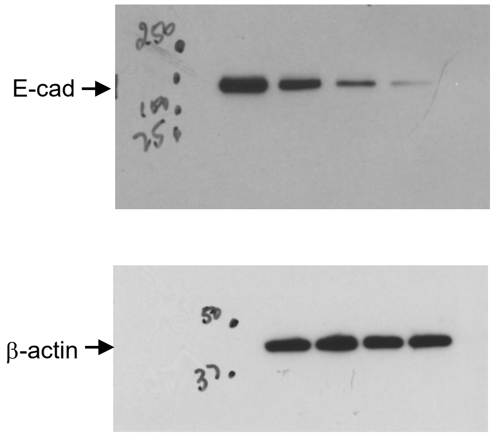

Supplementary figure 5G

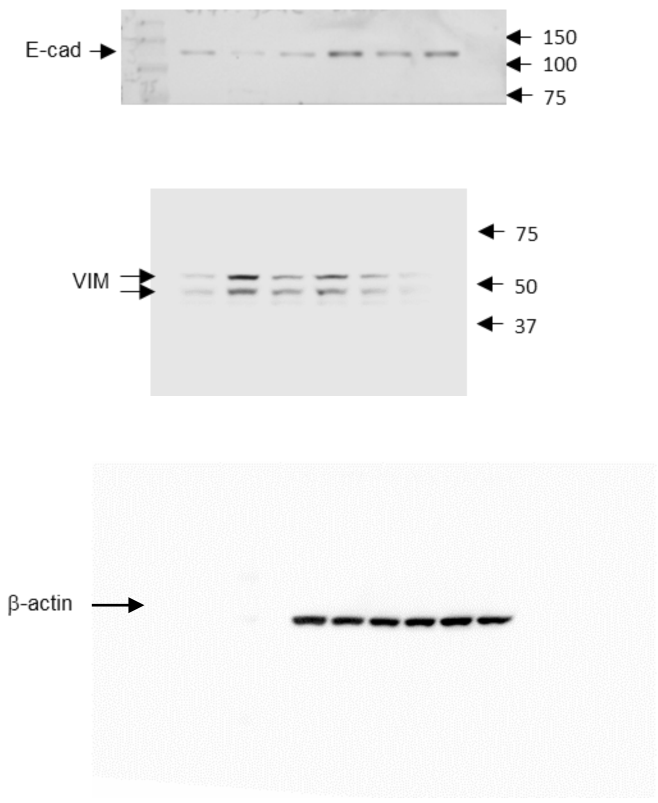

Supplementary figure 5H

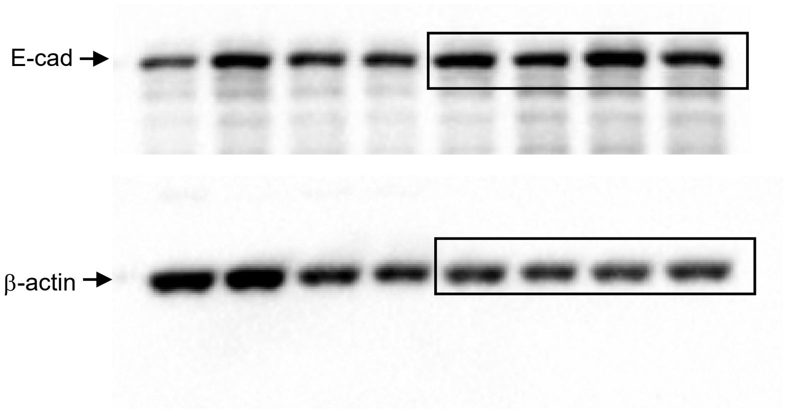

Supplement: Supplemental data [file jci-132-158096-s126.pdf]
